# Ketocarotenoid production in tomato triggers metabolic reprogramming and cellular adaptation: The quest for homeostasis

**DOI:** 10.1111/pbi.14196

**Published:** 2023-11-30

**Authors:** Marilise Nogueira, Eugenia M. A. Enfissi, Elliott J. Price, Guillaume N. Menard, Eudri Venter, Peter J. Eastmond, Einat Bar, Efraim Lewinsohn, Paul D. Fraser

**Affiliations:** ^1^ School of Biological Sciences Royal Holloway University of London Egham Surrey UK; ^2^ Plant Sciences for the Bioeconomy, Rothamsted Research Harpenden UK; ^3^ Department of Aromatic Plants Newe Ya'ar Research Center Agricultural Research Organization Ramat Yishay Israel; ^4^ Present address: RECETOX, Faculty of Science Masaryk University Brno Czech Republic

**Keywords:** Metabolic reprogramming, cellular adaptation, redox control, homeostasis, ketocarotenoid, Tomato

## Abstract

Plants are sessile and therefore have developed an extraordinary capacity to adapt to external signals. Here, the focus is on the plasticity of the plant cell to respond to new intracellular cues. Ketocarotenoids are high‐value natural red pigments with potent antioxidant activity. In the present study, system‐level analyses have revealed that the heterologous biosynthesis of ketocarotenoids in tomato initiated a series of cellular and metabolic mechanisms to cope with the formation of metabolites that are non‐endogenous to the plant. The broad multilevel changes were linked to, among others, (i) the remodelling of the plastidial membrane, where the synthesis and storage of ketocarotenoids occurs; (ii) the recruiting of core metabolic pathways for the generation of metabolite precursors and energy; and (iii) redox control. The involvement of the metabolites as regulators of cellular processes shown here reinforces their pivotal role suggested in the remodelled ‘central dogma’ concept. Furthermore, the role of metabolic reprogramming to ensure cellular homeostasis is proposed.

## Introduction

The negative impact of climate change will continue to escalate with unchecked global CO_2_ emissions. Replacing petro‐chemical‐derived products with plant‐based alternatives will play an important role in achieving the Sustainable Development Goals adopted by the United Nations (United Nations Environment Programme, 2022). It is expected that applied scientific endeavours will underpin this paradigm change to our manufacturing. However, the underlying progenitor to resolving these challenges is fundamental in nature. For example, to harness the full potential of plant‐based systems, it is essential to define metabolic and cellular plasticity on a holistic level. Only through the acquisition of this fundamental knowledge will biosynthetic capacity extend to deliver biorenewables at commercially viable levels. Therefore, understanding how a plant cell adapts to metabolic perturbation and re‐establishes homeostatic conditions is a fundamental aspect that underpins biotechnology in all its manifestations.

Ketocarotenoids are an example of high‐value natural colourants and antioxidants used across multiple industrial sectors, including aquaculture where they are an essential feed additive. Although naturally present in some bacteria and algae, their present mode of industrial production uses chemical synthesis (Pfander *et al*., 1997). Total chemical synthesis used in this case has adverse environmental credentials, as the process uses petro‐chemical‐derived precursors and rare metal catalysts (Bonrath *et al*., 2007). Hence, numerous examples exist whereby the formation of ketocarotenoids has been engineered into plant‐ and microbial‐based systems (Rodriguez‐Concepcion *et al*., 2018). The generation of a high ketocarotenoid tomato line and demonstration of its production, technical and economic feasibility are an exemplar of a sustainable plant‐based renewable source of ketocarotenoids for aquaculture (Nogueira *et al*., 2017). It was estimated that all the ketocarotenoid feed supplements required annually for aquaculture could be generated from this ketocarotenoid tomato genotype grown in 15 000 ha of greenhouse (Napier *et al*., 2020). Beyond its value as a biobased platform, the ketocarotenoid tomato is a fundamental resource for the study of cell plasticity, whereby complex biochemical and cellular adaptations in response to non‐endogenous metabolite production can be revealed.

The ketocarotenoid line was previously generated by crossing a low‐producing ketocarotenoid line (ZW), overexpressing the *Brevundimonas* β‐carotene hydroxylase (*CrtZ*) and β‐carotene ketolase (*CrtW*) (Enfissi *et al*., 2019) with an orange fruited recombinant inbred line accumulating a high level of β‐carotene, the main precursor of the ketocarotenoid biosynthetic pathway (Nogueira *et al*., 2017). This particular RIL was created from a genetic cross between the cultivated tomato *Solanum lycopersicum* with the orange fruited *Solanum galapagense* (Goldman *et al*., 1995; Paran *et al*., 1995). Within this population, the selection for orange fruit colour reflected the high expression level of the fruit ripening enhanced lycopene β‐cyclase (*Cyc‐b*), leading to high levels of β‐carotene.

In this study, the ketocarotenoid line was compared to the β‐carotene line (its orange fruited segregant), which resulted from the segregation of the *CrtZ* and *CrtW* genes, and to the control line, which is the wild‐type segregant. The β‐caro and keto lines both harboured the highly expressed *Cyc‐b* from *S. galapagense*. Tomato fruit at several developmental and ripening stages as well as leaf have been analysed using an integrated multi‐omic approach to identify holistic changes across metabolism. Hierarchical metabolomics from untargeted (LC‐QTOF‐MS) to multi‐platform analysis/quantification coupled to transcriptomics (RNA‐seq) has been performed on identical tissue. These data sets have been subjected to correlation network analysis. Subsequent validation of plastid changes has been carried out by transmission electron microscopy (TEM) and subchromoplast fractionation techniques. This system approach revealed the diverse range of adaptation mechanisms harnessed by the plant cell to facilitate the heterologous biosynthesis and storage of ketocarotenoids in the plastid membranes without inducing pleiotropic effects. Homeostasis is described as the self‐regulatory processes deployed by an organism, tissue or cell to maintain internal stability in response to external changes (Billman, 2020). Here, we discuss this concept in the context of internal changes occurring from the production of non‐endogenous molecules in cells.

Furthermore, these findings have been put into perspective with respect to the fundamental knowledge of cellular flux. The original central dogma of molecular biology, first presented by Francis Crick in 1957, described the transfer of information during DNA replication, transcription into RNA and translation into protein (Crick, 1958, 1970). For decades, metabolism has been overlooked in the central dogma (Schreiber, 2005) until advances in metabolomics shed light on the role of metabolites as fine and coarse regulators of biological systems (Kochanowski *et al*., 2017; Luca *et al*., 2020; Yang *et al*., 2018). This prompted the central dogma concept to be reviewed (Costa Dos Santos *et al*., 2021). This study supports the pivotal role of metabolites in the remodelled central dogma concept.

This study has used ketocarotenoid production in plants as an exemplar and utilized modern omic approaches to provide an insight into the metabolic reprogramming associated with providing the plant cell with the plasticity for high‐level production. The implications of the data in the future design of engineering biology strategies to produce valuable chemicals are discussed.

## Results

### Changes in chlorophylls, carotenoids and their esterification in the engineered tomato lines

**Figure 1:**
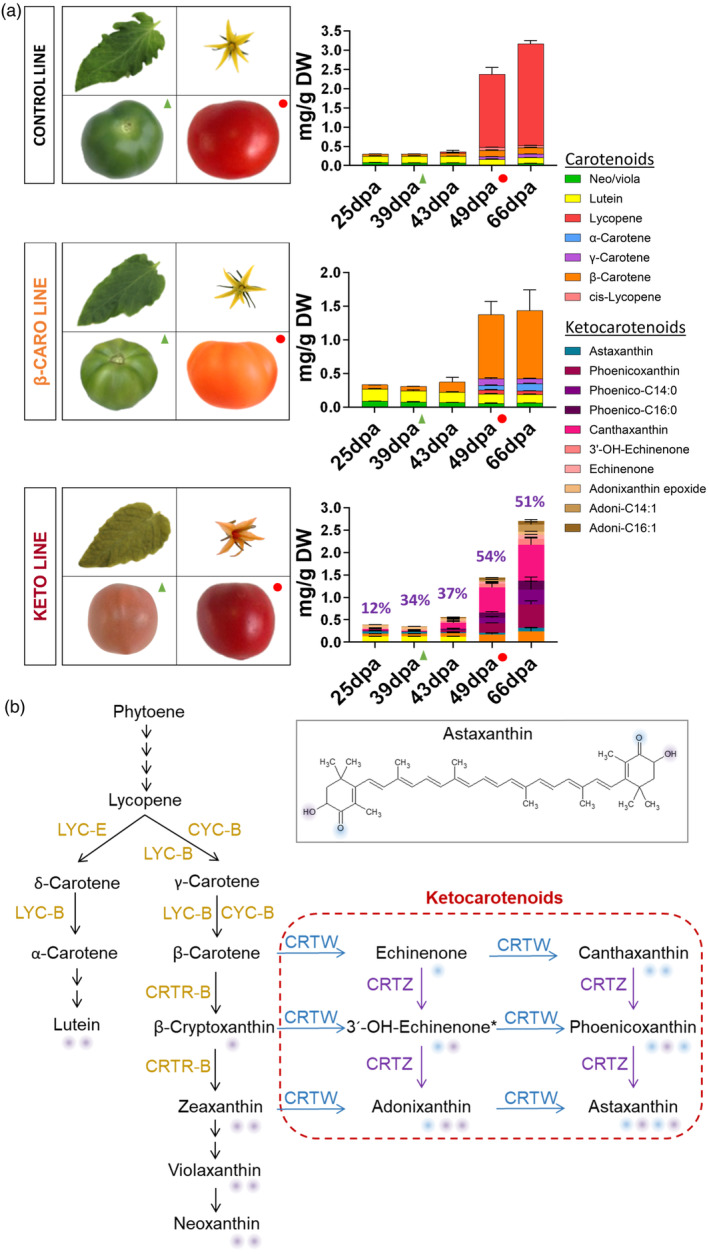
Tomato plant phenotypes and keto/carotenoid composition at five fruit ripening stages. (a) Phenotype of the leaf, flower, mature green fruit (39 dpa) and ripe fruit (49 dpa) of the control, β‐carotene (β‐caro) and ketocarotenoid (keto) lines and their fruit keto/carotenoid composition (only pigmented compounds are displayed) at five ripening stages (25, 39 (green triangle), 43, 49 (red circle), 66 days post anthesis). Data represent mean in mg/g dry weight ± SD. The rate of esterification of the main ketocarotenoid, phoenicoxanthin, is shown as % in the graph of the keto line. Astaxanthin esters data were not included; Neo, neoxanthin; Viola, violaxanthin; Phoenico, phoenicoxanthin; Adoni, adonixanthin. (b) Biosynthetic pathway of the endogenous carotenoids and heterologous ketocarotenoids (red rectangle) introduced in tomato plant. Enzyme names are as follows: LCY‐E, ε‐lycopene cyclase; LCY‐B, β‐lycopene cyclase; CYC‐B, fruit ripening enhanced β‐lycopene cyclase; CRTR‐B1, carotene β‐hydroxylase 1; CRTW, bacterial β‐carotene ketolase; and CRTZ, bacterial β‐carotene hydroxylase (*Brevundimonas* sp.). The purple and blue shadings depict the position of the functional hydroxyl and ketone groups, respectively.

The ketocarotenoid line, the β‐carotene line and the control line had very distinct fruit phenotypes, which reflected their carotenoid composition (Figure 1a). From 49 days post anthesis (dpa), the red fruit of the control line and the orange fruit of the β‐carotene line predominantly accumulated lycopene (red pigment) and β‐carotene (orange pigment), respectively (Figure 1a; Table S1). In tomato fruit, the lycopene cyclase enzyme (CYC‐B) is responsible for the conversion of lycopene to β‐carotene (Figure 1b). As described previously (Alba *et al*., 2005; Ronen *et al*., 2000), its expression decreased at 43 dpa, which resulted in an accumulation of lycopene in the control fruit (Figure 1a). However, in the β‐carotene line, the fruit ripening enhanced lycopene cyclase gene high expression (*Cyc‐b*, ~15‐fold increase compared to the control *Cyc‐b* at 49 dpa, Figure S1) led to high levels of β‐carotene (Figure 1a). From early development (25 dpa), the ketocarotenoid line accumulated a complex mixture of ketocarotenoids (deep red pigments), in free and esterified forms (Figures 1a and S2). Total ketocarotenoid content reached 2.5 mg/g DW in the 66 dpa keto fruit (Figure 1a; Table S1). The constant presence of a range of ketocarotenoids is explained by the constitutive expression of the heterologous *CrtZW* genes and the partial conversion of ketocarotenoid precursors to astaxanthin, the end product of this pathway (Figures 1b and S2). Only ketocarotenoids harbouring a hydroxyl moiety, such as adonixanthin, phoenicoxanthin and astaxanthin, can be esterified (Figure 1b). Those ketocarotenoids have all been found in their esterified form in the fruit (Figures 1a and S2; Table S1). The predominant ketocarotenoid, phoenicoxanthin, accounted for almost half of the ketocarotenoids quantified at 66 dpa. Esterification of phoenicoxanthin increased over fruit development from approximately 10% to 50% (Figures 1a and S2; Table S1). The ketocarotenoid fruit also differed by its chlorophyll content, which decreased (2‐ to 3‐fold) compared to the control and the β‐carotene line (Figure S3). The constitutive expression of *CrtZW* resulted in the presence of ketocarotenoids in the flowers and leaves of the ketocarotenoid line, leading to observable orange coloured petal and dark green to brown leaf hue (Figure 1a). In the leaf, similarly to the fruit, a complex ketocarotenoid profile was observed and the chlorophyll content was decreased (~1.5‐fold) compared to that in the control and β‐carotene lines (Figure S4a). However, contrary to the fruit where the production of β‐carotene was boosted due to a highly expressed fruit lycopene cyclase (*Cyc‐b*, Figure S1), in leaf, the production of ketocarotenoids depleted endogenous precursors, such as β‐carotene (2.5‐fold lower relative to controls) as well as other endogenous carotenoids (Figures S4a and 1). The efficiency of the photosystem II, which is reflected by the Fv/Fm value (the maximum photochemical efficiency of PSII in the dark‐adapted state), was also shown to be negatively affected (16% significant reduction, Figure S4b). Despite these changes in photosynthetic efficiency, no observable phenotypic effects besides altered colour were apparent.

### Ultrastructural changes to plastid structures resulting from altered carotenoid content

**Figure 2:**
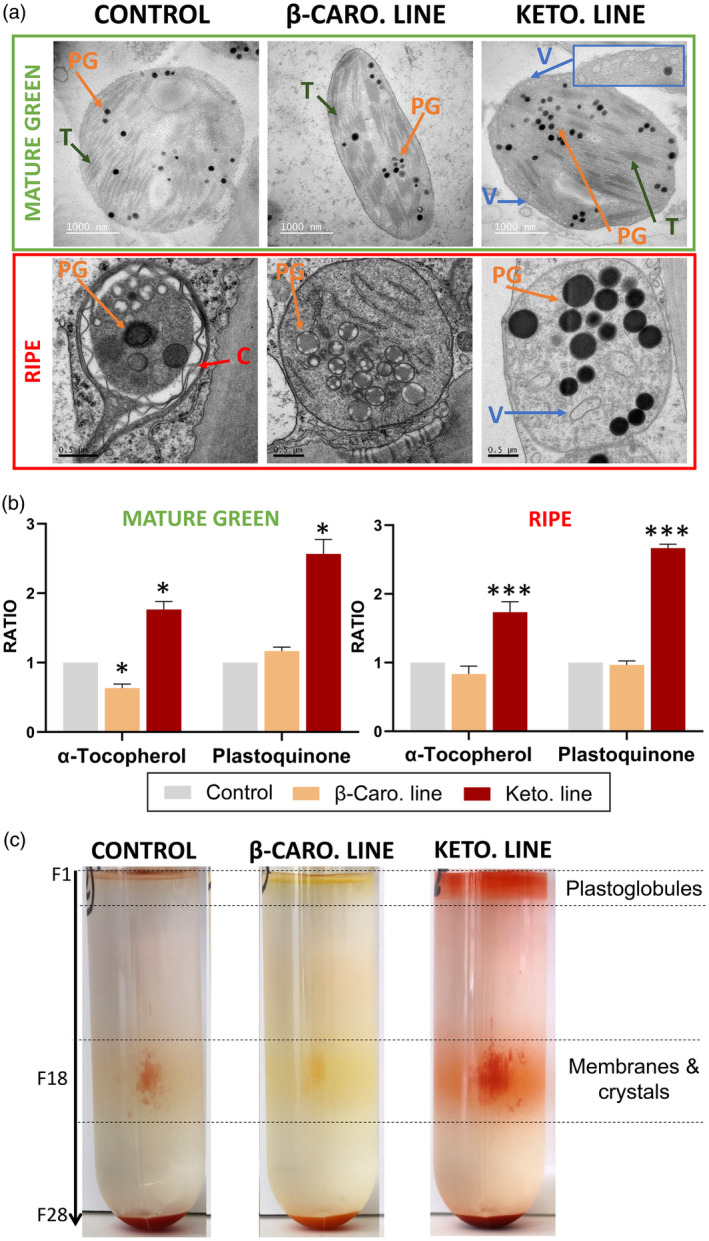
Ketocarotenoid sequestration and its effect on subplastidial compartment structures. (a) Electron micrographs of chloroplasts (mature green fruits, 39 dpa) and chromoplasts (ripe fruits, 49 dpa) of the control, β‐carotene (β‐caro) and ketocarotenoid (keto) lines. T, thylakoid; PG, plastoglobule; V, vesicles also called membranous sacs; C, lycopene crystal. The blue insert is a zoom in (2X) on the chloroplast envelope and its vesicles. (b) Fractionation of subchromoplast compartments of red fruit (~ 47 dpa) of the control, β‐carotene and ketocarotenoid lines. Each fraction (F) corresponds to 1 mL. (c) Alpha‐tocopherol and plastoquinone contents in the mature green and ripe fruit of the control, β‐carotene and ketocarotenoid lines. Data represent the ratio of the means compared to the control mean ± SD. Asterisks indicate significant differences compared to the control line (ANOVA). *P* < 0.05, *P* < 0.01, and *P* < 0.001 are designated by *, ** and ***, respectively.

Electron micrographs (EM) of the plastids were analysed to study the structural impact of the synthesis and storage of the ketocarotenoids on the cell. At mature green stage, the chloroplasts of the different lines looked similar, all presenting characteristic chloroplast sub‐compartments (thylakoid membranes and small plastoglobules). However, some differences were observed in the chloroplasts from the keto line compared to those of the other lines. The number of vesicles, which are derived from the chloroplast inner envelope membrane (Nogueira *et al*., 2013), was increased in the keto chloroplasts (Figure 2a). Almost 50% of the keto EM showed chloroplasts containing more than 10 vesicles compared to only 6% of the control EM (Table S2a). At ripe stage, as expected, the chloroplasts were converted to chromoplasts, the thylakoid membranes disintegrated, and the plastoglobules were of greater size. The chromoplasts of the different lines showed striking changes (Figure 2a). The plastoglobules in the keto chromoplasts represented a significant greater area (1.6 μm^2^) compared to the ones in the control line (0.3 μm^2^). The difference was due to an increased plastid area as well as a greater plastoglobule number in the keto chromoplasts (Table S2b). The plastoglobules of the keto chromoplasts also displayed a significant darker colour intensity (grey ratio of 4.4) compared to the plastoglobules of the other lines (grey ratio of 2.2). The same trend was observed at mature green (Table S2b). Compounds, such as plastoquinone and α‐tocopherol, are expected to contribute to the osmiophilicity of the plastoglobules (electron‐opaque/darkness) thanks to their ethylenic double bonds, which reduce the TEM staining agent OsO4 (Merriam, 1958; Van Wijk and Kessler, 2017). Both compounds were found in significantly greater levels in the keto chromoplasts compared to their levels in the control and β‐carotene chromoplasts (Figure 2b). Consequently, their increased contents correlated with the darker plastoglobules observed in the EM.

To investigate the subplastidial localization of the ketocarotenoids in the different lines, sub‐chromoplast compartments were separated on a sucrose gradient. Ketocarotenoids were found in the membranes, mainly in crystalline form, and in the plastoglobules of the keto chromoplasts, while the predominant carotenoids in the other lines, lycopene and β‐carotene were observed in the membranes and some also in crystalline form (Figure 2c; Table S3). It is worth noting that although β‐carotene is a cyclic carotenoid like the ketocarotenoids, only ketocarotenoids were stored in the plastoglobules (content greater than the residual baseline of the sucrose gradient, Figure S5). Therefore, a differential accumulation of carotenoids in the plastoglobules was observed despite similarities in chemical structures. The esterified ketocarotenoids, like their free form, were also found in both the membranes and plastoglobule fractions (Figure S5). The (keto)carotenoids (i.e. carotenoids and ketocarotenoids), which contain unsaturated double bonds, are also expected to affect plastoglobule osmiophilicity. Therefore, the ketocarotenoid localization also correlated with the darker plastoglobules observed in the EM of the keto chromoplasts.

### Holistic changes to metabolism beyond the formation of carotenoids

To study how the cell metabolism adapted in response to the ketocarotenoid production, a wide range of metabolites using multiple platforms that are typically suited to different compound classes were analysed (Tables S4 and S5). For example, amino acids, sugars, organic acids and carboxylic acids were analysed by GC–MS, volatiles by SPME‐GC–MS, carotenoids and isoprenoids predominantly by UPLC‐DAD, phenolics (LC‐QTOF‐MS) and phytohormones by LC‐QQQ‐MS. In addition, LC‐QTOF‐MS was also used for untargeted chemical fingerprinting using molecular features. A principal component analysis (PCA) integrating all metabolite data measured in ripe tomatoes, except the (keto)carotenoids data, revealed that all the biological replicates of each line formed independent clusters and that the greatest metabolic difference was observed between the keto line and the other lines (Figure S6). This result was representative of the PCA plots obtained for each analytical platform separately (i.e. LC‐QQQ‐MS, LC‐QTOF‐MS, GC–MS, SPME‐GC–MS, UPLC‐DAD). This highlighted that the alteration of the keto line metabolism affected compounds beyond the carotenoid pathway and that the variation between the keto line metabolism and the other lines was greater than the variation observed between the metabolism of the control and β‐carotene lines.

**Figure 3:**
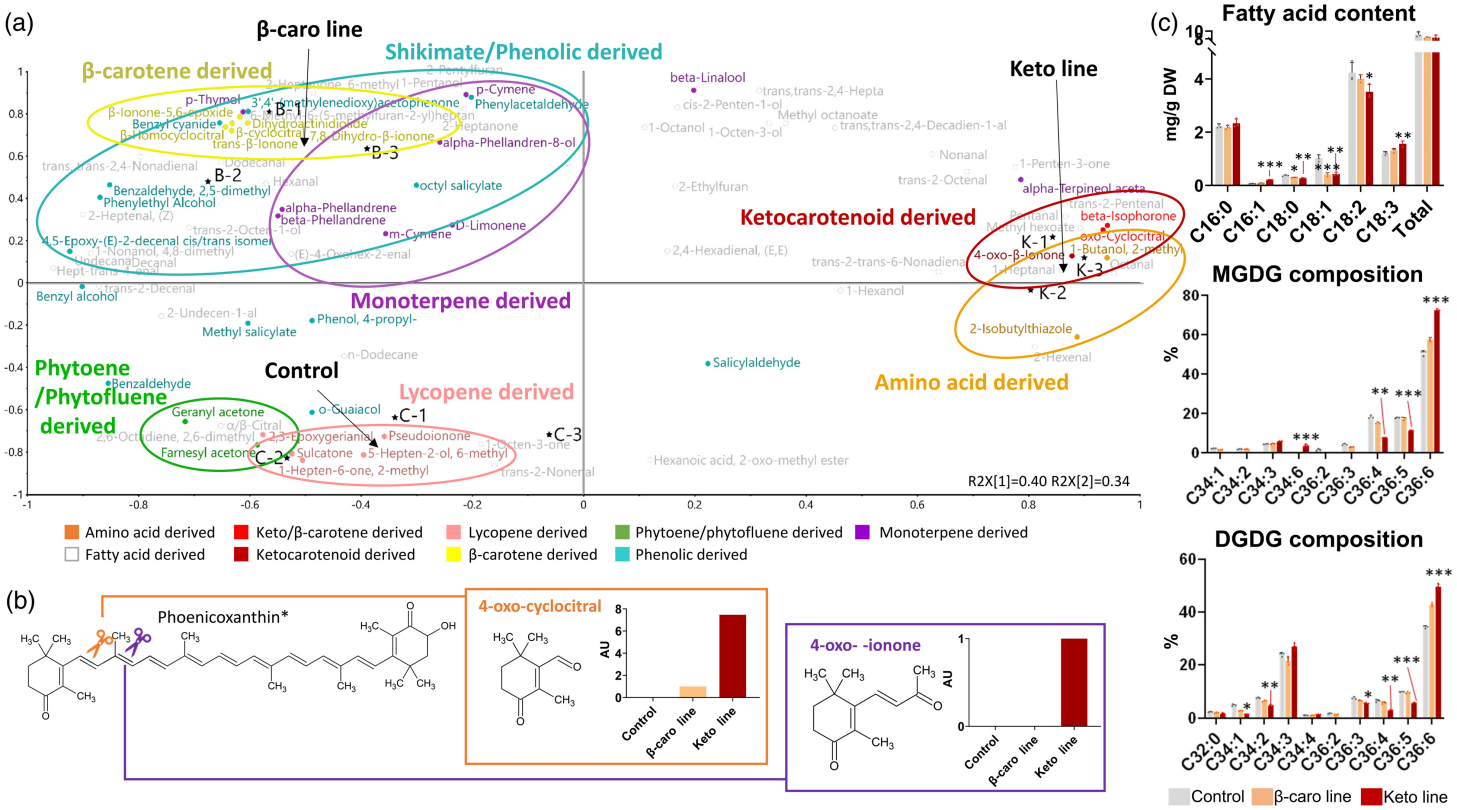
Effect of ketocarotenoid production on volatile and fatty acid contents in ripe tomato fruit. (a) Loading plots of principal component analysis of volatiles measured in ripe fruit of the control, β‐carotene (β‐caro) line and/or ketocarotenoid (keto) line. Volatile data are listed in Table S4. (b) Formation of the ketocarotenoid derived volatiles. The abundance of the volatiles in the control, β‐carotene line and/or ketocarotenoid line is displayed in bar charts; AU, arbitrary unit; *, phoenicoxanthin or any 4‐oxo ketocarotenoid. (c) Fatty acid content and plastidial lipid (MGDG, DGDG) fatty acid composition in fruit. Data represent mean in mg/g dry weight ± SD. Asterisks indicate significant differences compared to the control line (ANOVA). *P* < 0.05, *P* < 0.01 and *P* < 0.001 are designated by *, ** and ***, respectively. *P*‐values are listed in Table S13. MGDG, Monogalactosyldiacylglycerol; DGDG, digalactosyldiacylglycerol.

The analysis of catabolism revealed striking differences in the volatile content of the ripe fruit from different lines (Figure 3a; Table S4). Each line contained carotenoid‐derived volatiles, which corresponded to the cleavage of predominant (keto)carotenoid(s) in the fruit. For instance, (i) β‐cyclocitral and β‐ionone, β‐carotene‐derived volatiles, were found in proportionally greater amounts in the β‐carotene line; (ii) sulcatone and epoxygeraniol, lycopene‐derived volatiles, in the control line; and (iii) oxo‐β‐cyclocitral and oxo‐β‐ionone (non‐endogenous) in the keto line. Some volatile levels were increased up to 20‐fold and oxo‐β‐ionone was uniquely observed in the keto line (Figure 3b). A PCA of the volatile data revealed that the three tomato lines were not only characterized by their respective (keto)carotenoid volatiles but also, (i) for the β‐carotene line, by its phenolic/shikimate and monoterpene‐derived compounds and, (ii) for the ketocarotenoid line, by the branched‐chain amino acid (leucine and valine)‐derived volatiles. The composition of fatty acid derived compounds was different in all three lines. It is interesting to note that, penten‐3‐one, which derives from the C18:3 fatty acid, was increased by 2.5‐fold in the keto line (Table S4).

The alteration of the volatile profiles suggested changes in the cellular metabolism; therefore, the fatty acid content in the ripe tomato was examined. The total fatty acid content was not significantly different between the three lines; however, the fatty acid composition of the ripe keto tomato was shifted towards the synthesis of fatty acid with increased unsaturation (Figure 3c). Indeed, the content of C18:0, C18:1 and C18:2 was significantly decreased, while C18:3 level was increased by 1.3‐fold compared to that in the control line (Table S4). Moreover, an increase of the C16:1 content (3‐fold) was also observed in the keto line. Similarly, the fatty acid composition of the MGDG and DGDG lipids, which are structural lipids of the plastid membranes, also showed an increased in the percentage of C36:6 (C18:3 + C18:3) at the expense of the less unsaturated lipids C36:3 to C36:5 (Figure 3c). It is interesting to note that C34:6 (C18:3 + C16:3) was only detected in the MGDG analysis of the keto tomatoes.

**Figure 4:**
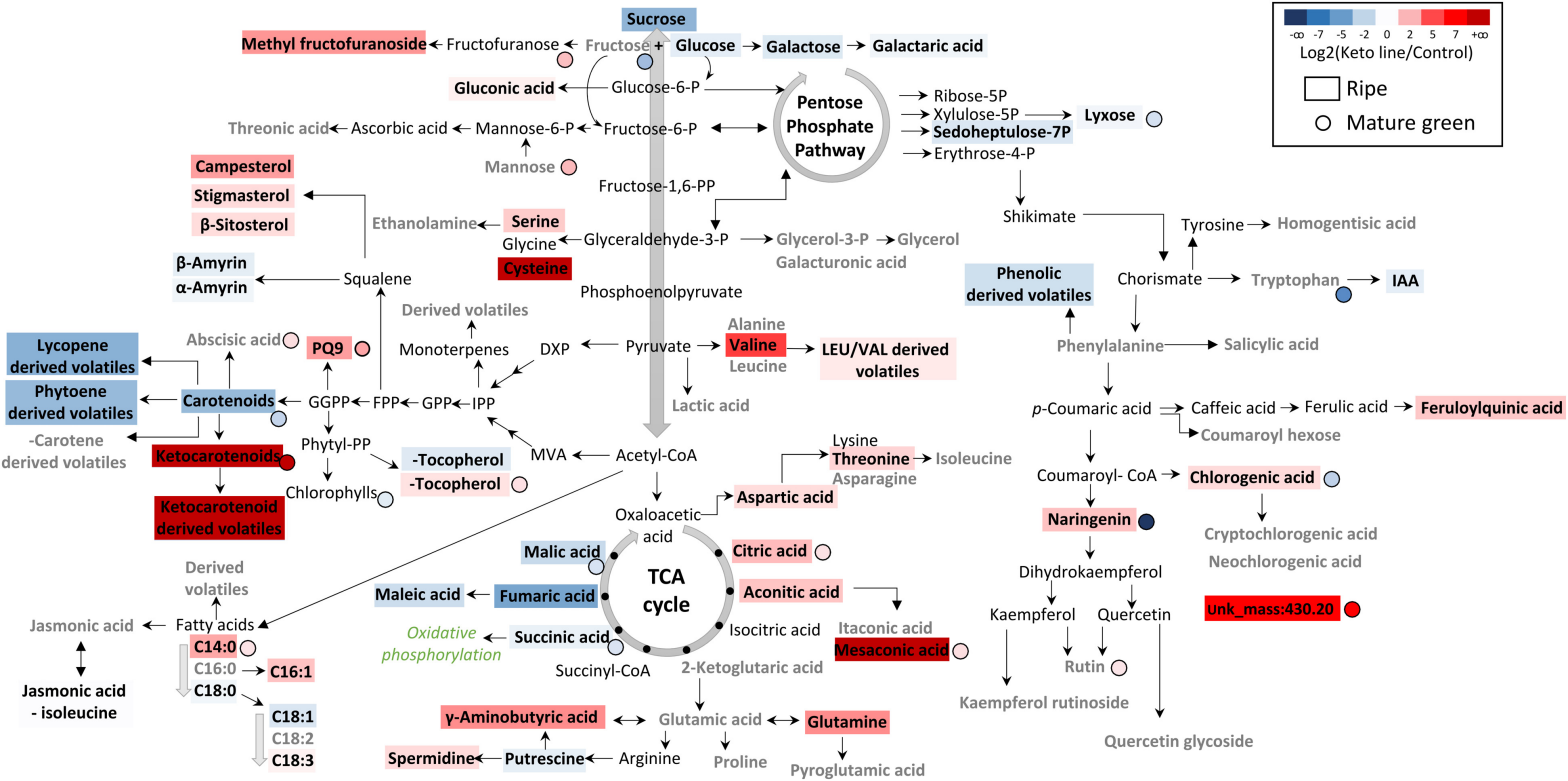
Biochemical pathways displaying the fold change metabolite levels in ripe and mature green fruit of the ketocarotenoid line compared to the control. Data represent the log2 of the ratio of means (keto line/control), and it is displayed by a colour illustrating a decrease (blue) or increase (red) of metabolite level in the keto line compared to the control. Compounds detected in the analytical platforms are shown in bold. The absence of significant difference is depicted by a grey bold font (t‐test, *P* > 0.05). Compounds showing a significant difference (black bold font) were attributed a colour representative of fold change. Metabolite levels quantified in ripe and mature green fruits are represented by a rectangle and a circle, respectively. The statistical significance of the volatile data in this display corresponds to the total content per class of compounds. The individual differences within the class of compounds are not shown. Metabolite data were compiled in Tables S4 and S5. *P*‐values are listed in Table S13. PQ9, plastoquinone 9; IAA, indole‐3‐acetic acid.

Ratios of metabolite levels in the keto line relative to the control were displayed on a pathway (Figure 4). The keto ripe fruit contained decreased levels of sugars such as sucrose, fructose and galactose and increased levels of amino acids (valine, cysteine and serine), phytosterols (campesterol, stigmasterol and β‐sitosterol) and phenolics (naringenin, chlorogenic acid and feruloylquinic acid). The levels of some unknown compounds, such as the unknown designated unk_mass:430.2, were increased by more than 100‐fold in the keto line. The changes in volatile contents often correlated with those of the metabolite levels from which they were derived. For instance, the levels of valine and valine derived volatiles were both increased in the keto line. Minor changes in phytohormone levels were observed such as, at MG, an increase in abscisic acid and, at ripe, an increase in jasmonic acid and a decrease in auxin. The levels of TCA cycle metabolites were consistently altered at MG and ripe, with an accumulation of citric acid, aconitic acid and mesaconic acid and a decrease of succinic acid, fumaric acid and malic acid (Figure 4; Tables S4 and S5).

The high production of β‐carotene in tomato fruit also led to global metabolic changes (Figure S7). Similarly to differences observed in the keto ripe fruit, the levels of some TCA cycle metabolites were also different at both MG and ripe in the β‐caro fruit compared to the control (decrease of malic acid and fumaric acid levels). However, in general, fewer pathways were altered compared to those in the keto/control line comparison (Figures 4 and S7).

Comparably to the ripe stage, the control, β‐caro and keto lines could also be differentiated by their metabolic profiles at MG independently of their carotenoid content (Figure S8a). The metabolic profile of the keto fruit was characterized by its content in carboxylic acids and phytohormones, while the profile of the β‐caro fruit was distinguishable by its elevated amino acid content and high levels of jasmonic acid and jasmonic acid‐isoleucine, and finally, the control line was differentiated by its phenolic and fatty acid contents (Figure S8b; Table S5).

### Impact of the ketocarotenoid production at a transcriptomic level

**Figure 5:**
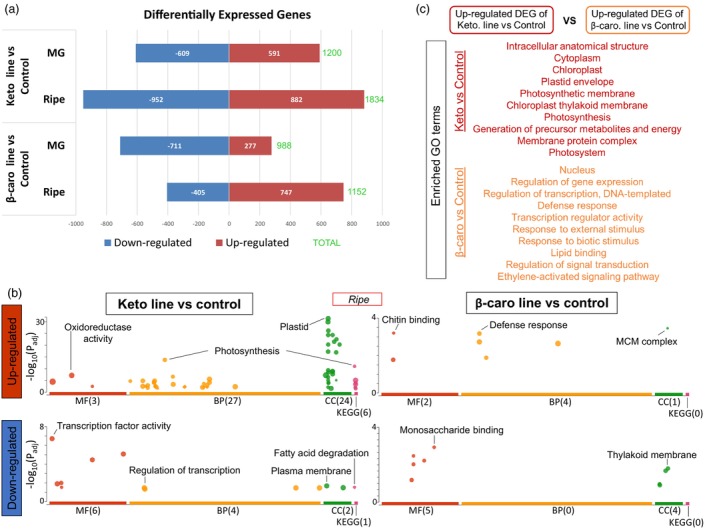
Differentially expressed genes and enrichment analysis of the transcriptomic data comparison of the tomato lines. (a) Up‐regulated and down‐regulated differentially expressed genes (DEG) between the ketocarotenoid and β‐carotene lines compared to the control, respectively, at mature green (MG) and ripe fruit stage. (b) Enriched Gene Ontology (GO) terms of the up‐regulated and down‐regulated DEG sets of the ketocarotenoid and β‐carotene lines versus control comparison, respectively. MF, molecular function; BP, biological process; CC, cellular component; KEGG, KEGG pathway. (c) Gene enrichment analysis of the up‐regulated DEG obtained from the ketocarotenoid line versus control comparison with the up‐regulated DEG from the β‐carotene line versus control comparison. The full list of enriched GO terms can be found in Table S11.

The variation of the transcriptome between the ketocarotenoid line, β‐carotene line and control line was investigated in MG and ripe fruit. Thereafter, the differentially expressed genes (DEG) obtained from the RNA‐seq comparison of the keto and the β‐carotene lines compared to the control line are referred to as keto/control and β‐carotene/control DEG, respectively. In general, the numbers of DEG found at the ripe stage were greater compared to the ones at MG for all DEG sets (Figures 5a and S9a). The higher number of DEG in the ripe fruit was concurrent with the significant accumulation of the (keto)carotenoids (Figure 1a). Moreover, at both developmental stages, the keto/control DEG were greater, in terms of number, compared to the β‐carotene/control ones, 1834 vs 1152 at ripe and 1200 vs 988 at MG, respectively. This is reflected in the RNA‐seq dendrogram of the ripe fruit, which showed that the keto line was the most distinct compared to the other lines (Figure S10). Therefore, both transcriptomic and metabolomic data (Figures S10 and S6b) indicated that the alterations in the ripe keto fruit were the greatest compared to the control. At MG, β‐carotene/control DEG were evident and corroborated the findings from metabolomic analysis (Figures 5a and S7).

Gene Ontology (GO) enrichment analyses confirmed that, when ripe, the keto/control comparison harboured up‐regulated DEG with many more enriched GO terms compared to those in the β‐carotene/control DEG (60 vs 7, respectively) and also compared to those found in the down‐regulated DEG (13 and 9, respectively, Figure 5b). In the ripe fruit, the enriched GO terms of the up‐regulated keto/control DEG were related to plastid membranes, photosynthesis, the generation of precursor metabolites and energy, oxidoreductase activity, carbon metabolism, biosynthesis of secondary metabolites and response to hydrogen peroxide. The enriched GO terms in the down‐regulated keto/control DEG were mainly linked to transcription regulation and fatty acid degradation (Figure 5b; Table S6). At MG, a greater number of enriched GO terms were found in the down‐regulated keto/control and β‐carotene/control DEG (35 and 54, respectively) compared to those in the up‐regulated DEG analyses (9 and 4, respectively). The enriched GO terms of the down‐regulated keto/control DEG at MG were related to the photosynthetic electron transport chain and the cell wall, while the ones found in the up‐regulated keto/control DEG were linked, for instance, to the response to ABA and to oxygen containing compounds (Figure S11; Table S6).

**Figure 6:**
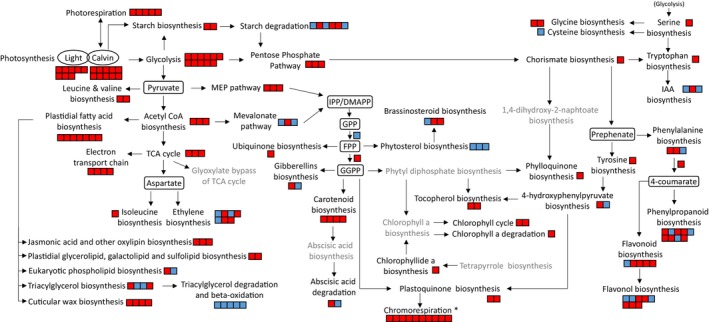
Transcriptional changes in the metabolic pathways of the ripe fruit of the keto line compared to the control. RNA‐seq data were used to create this display. Each square represents a gene of the referred pathway. Red and blue colours illustrate an increase and decrease in expression, respectively (positive or negative log2(Fold change)). The grey font denotes a lack of significant difference in expression level for all the genes investigated in the respective pathway (*P* > 0.05). All genes used to construct this figure are listed in Table S7 together with their log2(Fold change). Gramene database (www.gramene.org) and literature were used to compose the different metabolic pathways. IPP, Isopentenyl pyrophosphate; DMAPP, Dimethylallyl pyrophosphate; GPP, Geranyl pyrophosphate; FPP, Farnesyl diphosphate; GGPP, Geranylgeranyl pyrophosphate; IAA, Indole‐3‐acetic acid; TCA, Tricarboxylic acid cycle; * according to models proposed by Renato *et al*., 2014 and Grabsztunowicz *et al*., 2019.

The transcriptomic data of the ripe fruit have been displayed over biosynthetic pathways in Figure 6 (details in Table S7). The keto/control comparison revealed that genes of the carotenoid pathway were up‐regulated in the keto line, as well as genes encoding biosynthetic steps in associated pathways such as GGPP formation, supplying both the tocopherol and plastoquinone pathways (Figure 6). Up‐stream pathways providing carotenoid precursors, such as the MEP pathway, were also up‐regulated. Furthermore, core metabolic pathways such as the glycolysis, the TCA cycle, the pentose phosphate pathway and the photosynthesis, photorespiration, chromorespiration as well as electron transport chain were also positively regulated. Perturbations to the fatty acid and lipid biosynthetic pathway as well as phenolic formation were also observed. Therefore, the transcription has been altered not only for the carotenoid biosynthetic genes or closely related metabolic pathway genes but also at a global level whereby core metabolic pathway gene transcription was altered (Figure 6). The transcriptomic data of the β‐carotene/control comparison highlighted the up‐regulation of the transcription in the carotenoid pathway and down‐stream pathways such as ABA formation (Figure S12; Table S7). Alteration of transcription was also observed within the phenolic pathway and among core metabolic pathways but to a lesser extent compared to those seen in the keto/control comparison. Moreover, in the β‐carotene line, most changes in the core pathways were mainly down‐regulation of gene expression.

The endogenous β‐carotene level in the keto fruit seemed to have been maintained thanks to the changes in *Cyc‐b* gene expression. Indeed, *Cyc‐b* was up‐regulated by ~15‐fold in the ripe keto and the β‐carotene fruits compared to the control fruit and by ~4‐fold at MG but only in the keto fruit (Figure S1). On the other hand, the expression of the chloroplast lycopene cyclase *Lcy‐b*, which is responsible for the formation of β‐carotene in the MG fruit, was unchanged compared to the control in both tomato lines, and at both developmental stages studied (Figure S1). It was demonstrated earlier that the esterification of ketocarotenoids occurred from the early fruit developmental stage. PYP1 enzyme has been shown to esterify ketocarotenoids in ripe tomato fruit (Lewis *et al*., 2021). Here, the transcriptomic data revealed that *pyp‐1 esterase* expression was up‐regulated only at ripe (2‐fold) compared to the control, while at MG, there was another esterase, *gdsl esterase* that was up‐regulated (2.6‐fold). Altogether, this suggests that the adaptation to the presence of ketocarotenoids changed through development, since different enzymes (isoforms) were potentially recruited at different developmental and ripening fruit stages to deal with the occurrence of ketocarotenoid.

**Figure 7:**
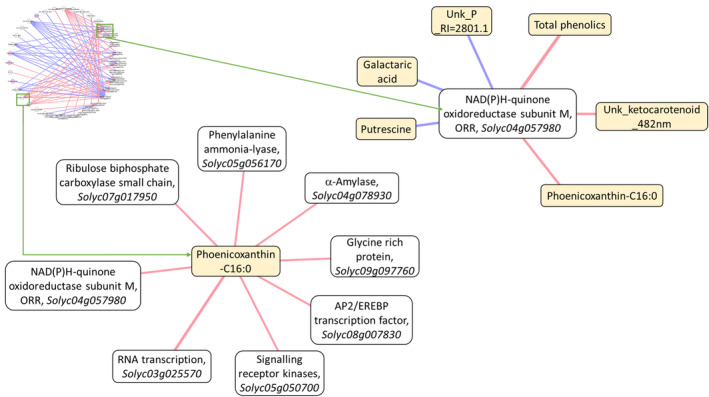
Gene‐metabolite correlation network analysis of the ripe keto line/control comparison. Metabolites and genes related to biosynthetic pathways, whose levels showed a statistical difference in the keto line compared to the control (*P* < 0.05), were selected to create the correlation network. Sub‐networks involving the ketocarotenoid phoenicoxanthin‐C16:0 and the gene NAD(P)H‐quinone oxidoreductase subunit M are shown separately. Metabolites and genes are represented in yellow and white rounded rectangle, respectively. Positive and negative correlations are denoted as pink and purple edges, respectively. The relative width of edges corresponds to the absolute value of the correlation coefficient. The Person correlation values are given in Table S9.

The chloroplastic malate dehydrogenase enzyme is considered a marker for the plastidial redox status (Nashilevitz *et al*., 2010; Obata *et al*., 2015). Its expression was up‐regulated (1.6‐fold) in the keto line compared to that in the control (Table S8). Similarly, many other genes related to redox control, such as thioredoxin, ferredoxin, cys‐peroxiredoxin and the chloroplastic cytochrome b6, were also up‐regulated (up to 3‐fold). In particular, the expression of the plastidial NAD(P)H‐quinone oxidoreductase subunit M (Ndh‐M) was increased by 20‐ and 11‐fold in the ripe keto fruit compared to that in the control and β‐carotene lines, respectively (Table S8). Ndh‐M protein is part of the NAD(P)H dehydrogenase complex (Ndh), which supports non‐photochemical electron fluxes from stromal electron donors to plastoquinones. In the chromoplast, this respiratory process can be referred to as chromorespiration, which is thought to take place within the vesicles or membranous sacs of the chromoplasts (Renato *et al*., 2014). The Ndh complex activity was proven to link redox activity to central and specialized metabolism such as carotenoid, tocopherol and phenolic metabolism (Nashilevitz *et al*., 2010). Here, a network analysis of the transcriptome and metabolome data of the keto/control comparison revealed that *Ndh‐M* was positively correlated to ketocarotenoids (*P*‐corr = 0.97 and 0.94) and to the content of total phenolics (*P*‐corr = 0.7) (Figure 7; Table S9). Moreover, one of the ketocarotenoids, phoenicoxanthin‐C16:0, was found to be positively correlated to, among others, *Ndh‐M* (*P*‐corr = 0.97), *rubisco* (*P*‐corr = 0.96), *phenylalanine ammonia‐lyase* (*P*‐corr = 0.96) and *α‐amylase* (*P*‐corr = 0.97) genes. This reinforces the idea that the ketocarotenoid biosynthesis is linked with core and specialized metabolism but also to redox control. Additionally, the expression of other Ndh subunits (B, L, N) was also all up‐regulated (up to 7‐fold) in the keto line (Table S8). Similarly, the expression of the gene coding for PsbQ‐like protein, which is required for the chloroplastic Ndh complex function (Yabuta *et al*., 2010) increased 15‐fold in the keto line compared to the control (Table S8).

In order to study the differences between the keto line and the control over ripening, GO enrichment analysis was carried out comparing the respective Ripe/MG DEG sets. It revealed that significantly less genes related to the GO terms ‘plastid’ and ‘photosynthesis’ had their expression level decreased over ripening in the keto line compared to those in the control, suggesting that plastid metabolism is, at least partially, kept on in the keto line over ripening (Table S10).

**Figure 8:**
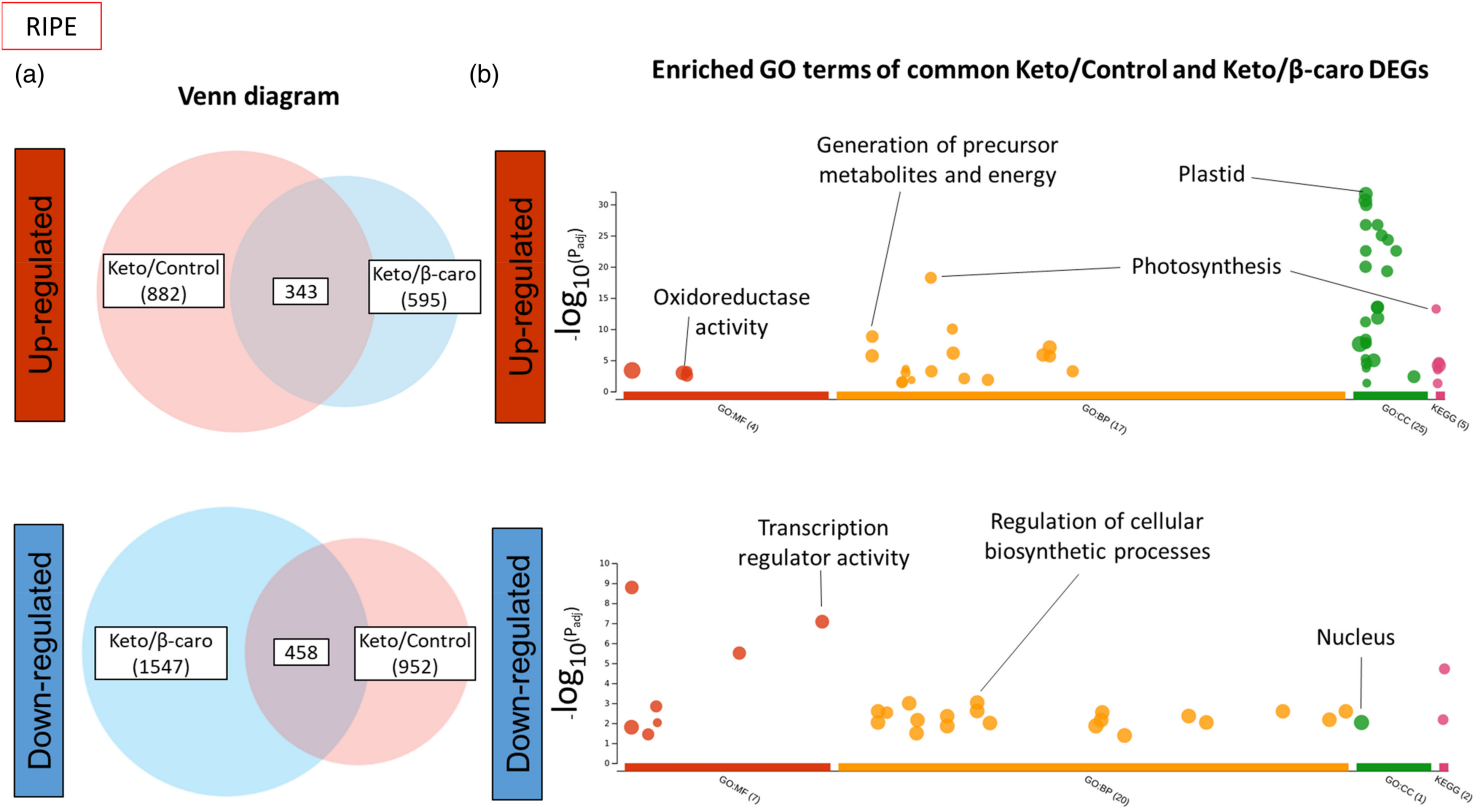
Gene enrichment analysis of the common DEGS of keto/control and keto/β‐caro sets in ripe fruit. (a) Venn diagrams of the up‐regulated and down‐regulated DEGs of the keto/control and keto/β‐caro sets. (b) Enriched GO terms of the up‐regulated and down‐regulated common DEGs between the keto/control and keto/β‐caro sets. MF, molecular function; BP, biological process; CC, cellular component; KEGG, KEGG pathway. The full list of enriched GO terms can be found in Table S12. Gene enrichment analysis was carried out with g:Profiler (https://biit.cs.ut.ee/gprofiler/).

Finally, to investigate how the cell, in the ripe fruit, was adapting differently when faced with the biosynthesis of the non‐endogenous ketocarotenoids compared to the biosynthesis of the endogenous β‐carotene, GO enrichment analysis was performed comparing the keto/control and β‐carotene/control up‐regulated DEG sets at ripe stage (Figure 5c; Table S11). The enriched GO terms for the adaptation to the ketocarotenoid production were linked to the plastid membranes, the photosynthesis and the generation of precursor metabolites and energy, while the enriched GO terms for the adaptation to β‐carotene production were mainly related to regulation of gene expression, regulation of primary metabolic process and response to stimulus. Similarly, to understand the characteristic responses caused by the formation and storage of the ketocarotenoids, GO enrichment analysis was carried out on the common keto/control and the keto/β‐carotene DEGs at ripe (Figure 8). The enriched GO terms in the common up‐regulated DEGs were associated with the plastid, photosynthesis, generation of precursor metabolite and energy and oxidoreductase activity, while those representing the down‐regulated DEG were connected to the nucleus, the transcription and regulation mechanisms of biosynthetic processes (Table S12).

Altogether, the transcriptomic data brought into light how the cells were adapting to the ketocarotenoids through modulating the expression of genes involved in the structure of the chromoplast, especially the membranes, the generation of precursor of metabolites and energy, the photosynthesis/chromorespiration and the redox control.

## Discussion

### Cellular and metabolic adaptation to the heterologous formation of ketocarotenoids

The tomato plant, engineered to produce high levels of valuable ketocarotenoids, is an exemplar to study how the cell is able to respond and adapt to the biosynthesis of non‐endogenous metabolites. In the present study, a series of potential adaptation mechanisms deployed by the cell have been highlighted. Firstly, the data suggested that structural adaptations to increase the storage capacity for the sequestration of ketocarotenoids arose in the chloroplast and chromoplast membranes. More vesicles (membranous sacs) and a greater number of darker (electron dense) and larger plastoglobules were found in the EM of keto plastids (Figure 2a,c). A diversification of storage locations was also observed. While the predominant carotenoids of the control lines were only found in the membranes (membranous sacs and thylakoid remnants), the free and esterified ketocarotenoids were also detected in plastoglobules. The boost in membrane surface area is most likely a response to the increase in metabolite levels such as the ketocarotenoids, α‐tocopherol and plastoquinone (Figures 1 and 2). Indeed, it was previously demonstrated that an increase in plastoquinone level led to enlarged plastoglobules (Szymanska and Kruk, 2010). Similarly, higher carotenoid levels resulted in altered membranous plastidial structures in many plant species (Berry *et al*., 2019; Cao *et al*., 2012; Enfissi *et al*., 2019; Fu *et al*., 2012; Kilcrease *et al*., 2013; Maass *et al*., 2009). Moreover, the plastid membrane composition was altered to presumably accommodate the ketocarotenoids. Indeed, the fatty acid composition of the main plastidial lipids (MGDG and DGDG) shifted from C36:3 to C36:6 (Figure 3c). The increase in the degree of unsaturation has been previously demonstrated as an adaptation mechanism to stresses such as cold temperature, whereby the lipid transition temperature is lowered to maintain membrane fluidity, in effect stabilizing the membranes (Guschina and Harwood, 2006; Leekumjorn *et al*., 2009; Menard *et al*., 2017; Wang *et al*., 2020). The esterification of (keto)carotenoids renders those metabolites more lipophilic, which favours their sequestration in membranous structures. In effect, this is also a mechanism to increase the carotenoid storage capacity of the plant cells (Torres‐Montilla and Rodriguez‐Concepcion, 2021; Van Wijk and Kessler, 2017). The esterification state of the ketocarotenoids did not influence their localization in the sub‐compartments of the keto chromoplasts (Figure S5). Secondly, adaptation mechanisms involving the recruitment of promiscuous enzymes (esterification and catabolism of the ketocarotenoids, Figure 3a,b; Table S7) and/or enzymes whose genes are expressed under less regulatory control at a specific developmental stage have also been witnessed. For example, the recruitment of the fruit *Cyc‐b* enzyme in the keto MG fruit instead of the favoured lycopene β‐cyclase (*Lcy‐b*, Table S7) at MG. Other examples of this adaptive mechanisms have been highlighted in the literature (Enfissi *et al*., 2017, 2019; Karniel *et al*., 2022; Mortimer *et al*., 2016, 2017; Nogueira *et al*., 2013). Thirdly, in order to cope with the synthesis of the ketocarotenoids, the core metabolic pathways across the cell have been mobilized for the generation of metabolite precursors and energy (Figures 4, 5, 6). In particular, the TCA cycle would seem to be providing reductants for mitochondrial respiration, fatty acid biosynthesis and possibly chromorespiration (Figures 4 and 6). The up‐regulation of the expression of genes involved in the chromorespiration could also be linked to the increase of membranous surface in the chromoplasts, as chromorespiration is believed to take place in vesicles (Renato *et al*., 2014). Consequently, changes in all sub‐compartments of the cell were observed. This suggests that regulatory mechanisms were in place to orchestrate those global changes.

### Redox control as a global orchestrator of cell adaptation

The plant's capacity to rapidly adapt to environmental cues relies principally on the quest for reduction–oxidation (redox) homeostasis at the cellular level. External stresses usually lead to a change in redox status, which is sensed, and redox regulation mechanisms are activated to restore homeostatic balance. The redox status plays a pivotal role in coordinating metabolic processes. Simultaneously, the redox status regulates itself through the metabolism with many metabolic pathways being targets of redox regulation. Consequently, the redox regulation of the metabolism results in modification of the redox status itself (Cejudo *et al*., 2019; Geigenberger and Fernie, 2014; Hernandez and Cejudo, 2021; Obata *et al*., 2015; Yokochi *et al*., 2021). In the present article, the plant cells were not reacting to external signals but to new intracellular cues in the form of non‐endogenous metabolites. As a result of the high‐level production of ketocarotenoids in the cell, a global response was initiated (Figures 4, 5, 6). The intertwined relationship of redox control and metabolism suggests that redox control must have played a part in orchestrating those global changes observed and was in turn self‐regulated by the redox changes occurring. The data in this article corroborated this hypothesis, as further described. For example, changes in redox related gene expression were observed (Figure 6; Table S8). It is also known that NAD(P) and NAD(P)H couples are integrated in most of the core metabolic processes such as the glycolysis, the tricarboxylic acid (TCA) cycle, the respiratory electron transport, the photosynthesis and the oxidative pentose phosphate pathway (oxPPP) (Obata *et al*., 2015). All the mentioned pathways have been altered at a transcriptomic and/or metabolic level (Figures 4 and 6). Moreover, the transcript of NAD(P)H dehydrogenase subunit M (*Ndh‐M*), which is part of a plastidial respiratory mechanism (Renato *et al*., 2015), was one of the most up‐regulated genes (20‐fold) in the ripe keto/control DEG. There was no difference in *Ndh‐M* expression at the mature green stage between the keto and control fruit. However, while, in the control, its expression decreased (16‐fold) through ripening, it remained constant in the keto fruit (Table S7). A similar trend was observed with other *Ndh* subunits (Table S8). Furthermore, the plastidial Ndh complex activity has been shown to impact central metabolism as well as antioxidant levels such as carotenoids, flavonoids and tocopherols (Nashilevitz *et al*., 2010). Hence, the changes observed in the ketocarotenoid tomato, namely the alteration of the core and specialized metabolism at metabolomic and transcriptomic level, suggest that the Ndh complex could have played an important role in the response of the redox control. Additionally, the carotenoid pathway itself is directly connected to the redox status with some of its enzymes, phytoene desaturase (PDS) and zeta‐carotene desaturase (ZDS), being part of the net electron transfer, while 1‐deoxy‐D‐xylulose 5‐phosphate reductoisomerase (DXR), lycopene β‐cyclase (LCYB), β‐carotene hydroxylase (CRTR‐B) and Zeaxanthin epoxidase (ZEP) requiring NADPH or high PQ/PQH2 for their reactions (Figure 1b, Fanciullino *et al*., 2014, Nashilevitz *et al*., 2010). Therefore, alterations within the carotenoid pathway, which were triggered by the biosynthesis of ketocarotenoids, ought to have disrupted the redox homeostasis. The non‐photochemical electron transport in the chromoplast, also called chromorespiration, is believed to involve, the Ndh complex, plastoquinone, the carotenoid enzyme PDS, PTOX, the cytochrome b6f and an ATP synthase (Grabsztunowicz *et al*., 2019; Renato *et al*., 2014). The plastoquinone pool is a redox sensor that regulates the redox status (Havaux, 2020). In particular, it has been shown in *Haematococcus* that it is a redox sensor for carotenoid biosynthesis (Steinbrenner and Linden, 2003). Moreover, active exchange of plastoquinone between the thylakoid membrane and the plastoglobules is important for tight environmental control (Havaux, 2020). In stress conditions, a redistribution of plastoquinone towards the plastoglobules occurs, possibly due to the increased occupancy in the membrane, which restrict plastoquinone diffusion (Kirchhoff, 2014). Here, our data showed an increased plastoquinone content in the keto tomato compared to the control line, which was previously hypothesized to be linked with the enlarged and darker plastoglobules observed in the keto EM (Figure 2). Moreover, hydrogen peroxide formation within the plastoquinone pool is thought to participate in retrograde signalling and in modulation of expression (Havaux, 2020). Hydrogen peroxide has also been suggested to play a key function in the control of the redox state of stromal enzymes (Cejudo *et al*., 2019) and signalling between cell sub‐compartments (Buchanan and Balmer, 2005). The enriched GO term ‘response to hydrogen peroxide’ in up‐regulated DEGs of the keto tomato compared to the control (Table S6) reinforced the participation of the redox control in the cell response to ketocarotenoids. In addition, antioxidants, such as tocopherols, are considered information‐rich redox buffers, which react with a myriad of cellular components (Shao *et al*., 2008). The interaction between ROS and antioxidant is believed to act as integrator of signals derived from the environment and the metabolism (Foyer and Noctor, 2005; Shao *et al*., 2008). Therefore, the increase in tocopherol content in the keto tomato had most likely an impact on redox signalling. Finally, the changes in TCA cycle metabolites studied (Figure 4) and the up‐regulation of the expression of the plastidial malate dehydrogenase (Table S7) indicated a possible alteration of the malate valve to maintain redox homeostasis between sub‐cellular compartments such as chloroplast and mitochondrion (Buchanan and Balmer, 2005; Selinski and Scheibe, 2019). Together, these lines of evidence corroborate the involvement of redox control as part of an adaptation mechanism to the biosynthesis and storage of the ketocarotenoids.

### The impact of the ketocarotenoid production on photosynthesis and beyond

In comparison with previous ketocarotenoid forming lines such as the ZW tomato line (Enfissi *et al*., 2019), the inclusion of the highly expressed fruit *Solanum galapagense* lycopene β‐cyclase (*Cyc‐b*) restored β‐carotene levels in the keto fruit studied (Figure 1; Tables S1 and S3). Interestingly, several differences were highlighted between the ZW line and the keto line (i) only the keto line could produce high levels of ketocarotenoids, due to the consistently maintained levels of the precursor β‐carotene in the fruit; (ii) the ZW chloroplasts resembled chromoplasts due to the lack of thylakoid structures, while the keto line had wild‐type chloroplasts, with the addition of more vesicles; (iii) the ripening of the ZW tomatoes was delayed compared to that in the control tomatoes, but no delay was observed in the keto tomatoes. Although the ZW tomatoes were producing 15 times less ketocarotenoids compared to keto tomatoes (Enfissi *et al*., 2019), the impact on the chloroplast sub‐structures and ripening was a lot more pronounced. This hints at the importance of β‐carotene in the global metabolism. Indeed, some carotenoids, including β‐carotene, are thought to be essential elements of photosynthesis. In the leaf, the Fv/Fm value (the maximum photochemical efficiency of PSII in the dark‐adapted state) has been negatively impacted by the presence of the ketocarotenoids in the chloroplast membranes (Figure S3). This has been recorded in other ketocarotenoid engineered plants (Enfissi *et al*., 2019; Mortimer *et al*., 2017; Xu *et al*., 2020). Ketocarotenoids have been found to replace some or all carotenoids in the light‐harvesting complexes (Liguori *et al*., 2017; Mortimer *et al*., 2017); nevertheless, it has been proven that the photosynthetic system was still functional (Xu *et al*., 2020). The process of photosynthesis has shown a high adaptive capacity by being able to remain functional with only ketocarotenoids as accessory carotenoid pigments and, for instance, by compensating for the decrease in PSII/PSI antenna size in the keto engineered tobacco by a high PSII/PSI ratio (Xu *et al*., 2020). In the fruit, alteration of photosynthesis has also been demonstrated at the transcriptomic level with the up‐regulation of photosynthetic genes (Figure 6) and the enriched GO terms linked to photosynthesis in the keto ripe tomato (Figure 5; Table S6). In particular, the expression of the genes encoding the curvature thylakoid proteins has been up‐regulated in the keto ripe fruit (Table S7). Those proteins have been suggested to play a role in the PSII repair cycle and in facilitating the connection between LHCII and PSI (Trotta *et al*., 2019). It is known that photosynthesis in the mature green tomato fruit is functional but is only fixing up to 20% of the carbon (Pesaresi *et al*., 2014; Simkin *et al*., 2020). Therefore, the fruits are still heavily dependent on the sugars provided by leaf photosynthesis (Lytovchenko *et al*., 2011). The photosynthesis in the keto leaf has been negatively impacted (Figure S4); similarly, it could have been affected in the keto fruit but to a lesser extend as the fruit contained wild‐type level of β‐carotene. Decreased sucrose content was found in the ripe keto tomato relative to the control (Figure 4; Table S4). At a transcriptomic level, the photosynthesis genes seemed to have been first down‐regulated in the MG keto tomato and then up‐regulated in the ripe keto tomato (Figure 6; Table S10). Rubisco (small unit), a main enzyme for the fixation of CO_2_ in the Calvin cycle, has been positively correlated (*P*‐corr = 0.96) to a ketocarotenoid in a network analysis (Figure 7; Table S9). Its expression in the control fruit drastically decreased through ripening (35‐fold), while in the keto fruit it remained constant (Table S7). It is possible that the photosynthesis in the fruit was compensating for the impacted photosynthesis in the struggling leaves. The photosynthesis compensation mechanism between plant compartments has already been shown. Indeed, the literature describes that any decrease in the rate of fruit photosynthesis can be compensated for by the up‐regulation of leaf photosynthesis and an increased import of photoassimilates from the leaves (Araujo *et al*., 2011; Nunes‐Nesi *et al*., 2005). The suggestion that the fruit photosynthesis could be up‐regulated to boost the metabolism has implication for future genetic engineering of sink organs to enhance yield, fruit size and nutritional quality (Simkin *et al*., 2020). Moreover, the data described here suggest that other respiratory mechanisms have also been up‐regulated to compensate for chemical energy synthesis such as chlororespiration and/or chromorespiration, which are both linked to, among others, NDH enzyme and plastoquinone levels (Figure 6). An extensive comparison of the ZW and keto tomato leaf and fruit would certainly shed more light on the plasticity of photosynthesis and other respiratory processes.

### Cell reprogramming or the quest for homeostasis

**Figure 9:**
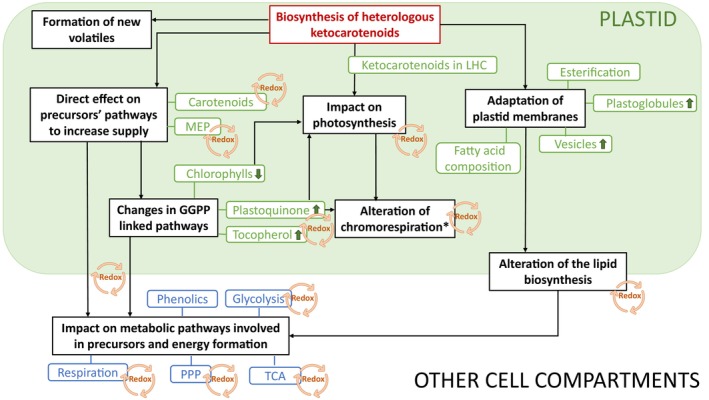
Transcriptomic, metabolic and cellular reprogramming triggered by the production of heterologous ketocarotenoids. MEP, Methylerythritol 4‐phosphate; GGPP, Geranylgeranyl pyrophosphate; PPP, Pentose phosphate pathway; TCA, Tricarboxylic acid cycle; LHC, Light‐harvesting complex. Green arrows indicate an increased level. *, according to models proposed by Renato *et al*., 2014, and Grabsztunowicz *et al*., 2019. See main text for figure description.

It is clear that in order to maintain cellular homeostasis, holistic system‐level changes arise in response to non‐endogenous ketocarotenoid production. Deciphering the precise sequence of events will require further validation. However, Figure 9 has outlined the changes with direct and indirect effects, illustrating a potential cascade of events. For example, (i) the targeted carotenoid pathway is perturbed and transcriptional regulation is induced to regulate the carotenoid pathway flux; (ii) related plastidial isoprenoids are effected potentially from ubiquitous precursor deviation; (iii) global changes across primary and intermediary metabolism arise, presumably to supply source precursors for complex energy‐consuming secondary metabolites; (iv) these metabolic changes, in the carotenoid pathway and beyond, are inevitably impacting the redox mechanism of the cell, which in turn is likely to drive secondary transcriptional and biochemical regulation; and (v) the change in metabolite composition is directing organelle adaptation in order to accommodate non‐endogenous metabolites. Another feature that is evident arises from enzyme promiscuity, highlighted by the formation of carotenoid esters and new volatiles not reported in the volatome of tomato to date.

In summary, our multi‐omic approach has gone beyond the target pathway to show the complexity of metabolic reprogramming events that arise in an attempt to maintain homeostasis. Knowing the metabolic limitations of the plant and how these constraints could be overcome will aid in the rationale design of future engineering and precision breeding approaches. Transcription factors have been used and are routinely proposed as the key to coordinated gene expression, for optimal flux through a pathway (Zhang *et al*., 2015). This is in line with the standard representation of the central dogma and its proposed flow of information. However, in the present study, it appears that the production of non‐endogenous metabolites is the initiators of global metabolic reprogramming, that employs multiple layers of cellular regulation, including transcription and organelle adaptation. Interestingly, in animal systems, cancerous tissues display metabolic reprogramming as a mechanism to attempt to maintain homeostasis, linked to the generation of energy. The metabolite changes observed in the ketocarotenoid producing lines are similar, whereby the nature of energy production, lipid and amino acid formation are perturbed, in addition to organelle biogenesis and macromolecular production occurring. Traditionally, these changes in cancerous tissues have been attributed to induction of transcription factors (Ell and Kang, 2013; Sanda *et al*., 2012). However, alterations in specific metabolism/metabolite levels have more recently been postulated as the progenitors of transcriptional master regulators (Martín‐Martín *et al*., 2018). The latter would appear to be the case in the present metabolic engineering study with non‐endogenous compounds, initiating a series of metabolic reprogramming events. This regulatory process across kingdoms warrants further study and highlights the need to consolidate the role of metabolites as a component of the central dogma (Costa Dos Santos *et al*., 2021; De Lorenzo, 2014).

## Methods

### Plant material and growth conditions

The ketocarotenoid, β‐carotene and control tomato (*Solanum lycopersicum*) lines were generated in previous studies (Enfissi *et al*., 2019; Nogueira *et al*., 2017) where they are referred to as ZWRI, ZWØRI and ZWØRIØ, respectively. The tomato plants were greenhouse grown (25 °C day/15 °C night) at Royal Holloway University of London (England), with supplementary lighting (16 h day/8 h night).

### Metabolite extraction and analysis

Freeze‐dried tomato material was homogenized to a fine powder with a tissue disruptor TissueRuptor (Qiagen). Extraction protocols varied for a minimum of three biological and three technical replicates and included one quality control and one extraction blank per randomized batch. Each biological replicate consisted of a pool of three fruits or leaves from one plant.

### Carotenoid, chlorophyll, and plastoquinone analyses

Carotenoids, chlorophylls, and plastoquinone were extracted from 15 mg of tomato powder as previously published in Nogueira *et al*. (2013). Metabolites of interest were separated and identified by Ultra High Performance Liquid Chromatography with photo diode array detection (UPLC‐PDA) as described in Nogueira *et al*. (2013) for plastoquinone and in Nogueira *et al*. (2017) for carotenoids and chlorophylls. A modified method of the latter was used to analyse the putative astaxanthin di‐esters, with the 0% A: 100% B step lasting for 11 min instead of 2 min. Carotenoids, chlorophylls and plastoquinone were quantified using dose–response curves made using authentic standards (Table S14). Identification of the ketocarotenoids esters was previously published in Nogueira *et al*. (2017). They were quantified using the dose–response curve of their corresponding free carotenoid. Content was expressed as μg/g DW.

### Fatty acid analysis

Fatty acids were quantified following the method detailed in Nogueira *et al*. (2017). Fatty acid composition of digalactosyldiacylglycerol (DGDG) and monogalactosyldiacylglycerol (MGDG) was analysed using electrospray ionization tandem triple‐quadrupole mass spectrometry (API 4000 QTRAP; Applied Biosystems; ESI‐MS/MS) as described in Menard *et al*. (2017) except that total lipids were extracted from 20 mg of freeze‐dried tomato powder.

### Primary/intermediary metabolism analysis

Tomato powder aliquots (10 mg) were extracted in 50% methanol (1 h, shaking, room temperature (RT)) followed by the addition of 1 volume of chloroform (500 μL). After centrifugation, the non‐polar extract was placed in a fresh centrifuge tube, while the polar phase was re‐extracted with 1 volume of chloroform. Polar (500 μL, methanolic epiphase) and non‐polar metabolites (total: 1 mL, organic hypophase) were collected, after centrifugation. Saponified non‐polar metabolites were obtained by modifying the protocol with the addition of 60% KOH (10X) in 50% methanol followed by an incubation at 42 °C for 1 h in a thermomixer (300 rpm Eppendorf), instead of the 1 h rotation at RT. Polar extract (10 μL) and non‐polar extracts (1 mL) were spiked with 10 μL of the internal standard solution (1 mg/mL of deuterated succinic acid in methanol and 1 mg/mL of deuterated myristic acid in chloroform, respectively) and dried under vacuum (Genevac EZ.27). Dried extracts were derivatized to their methoxymated and silylated forms as described in Nogueira *et al*. (2013). Gas chromatography–mass spectrometry analysis was performed on an Agilent 7890 (UK) gas chromatograph with a 5975C MSD as detailed in Almeida *et al*. (2021). Peak identification was conducted according to metabolomics reporting guidelines (Table S15). Peak areas were expressed relative to internal standard.

### Phenolics

Following the same extraction protocol as for primary metabolites, an aliquot of the polar extract was analysed on an Agilent 6560 Ion Mobility Q‐TOF coupled to an Agilent 1290 Infinity II (Agilent Technologies, Inc.) as described in Drapal *et al*. (2020). Peak areas were expressed relative to internal standard.

### Phytohormones

Isopropanol (Fisher Chemical) and water (2:1) with 0.1% HCl (500 μL, Fisher Chemical) were added to homogenized ripe tomato material (20 mg), and samples were vortexed (10 s) and rotated (1 h) at RT. Dichloromethane (Fisher Chemical) was added, and samples vortexed (10 s) and centrifuged (20 000 RCF, 5 min). Aliquots (100 μL) of the polar phase were transferred to glass inserts and dried under nitrogen. Samples were then resuspended in methanol (100 μL). Extracts were injected (1 μL) into a 1290 Infinity II UPLC (Agilent Technologies), coupled to a 6470 triple‐quadrupole (QQQ) MS (Agilent Technologies). The column, UPLC conditions, mobile phase and temperature were identical to the settings used for the flavonoid analysis. The solvent gradient was different. It started at 95% (A) for 1 min, followed by a linear decrease to 75% (A) at 3 min, 60% (A) at 6 min and 2% (A) at 8 min, which was held until 9.5 min before returning to initial conditions of 95% (A) at 10.5 min. The column was then re‐equilibrated for 5 min. Samples were simultaneously analysed in ESI+ and ESI− modes, with capillary voltage set to 3500 V and 4000 V, respectively, and nozzle voltage of 2000 V and −500 V, respectively. The N2 drying gas flow rate was 11 L/min, and the cell accelerator voltage was 5 V. A dynamic multiple reaction monitoring (MRM) method was used to selectively analyse the phytohormones of interest. Details for each phytohormone are given as retention time (min), scan mode, collision energy (V), fragmentor (V), precursor ion (m/z) and product ion (m/z): zeatin: 2.4 min, negative, 19 V, 80 V, 218.10 m/z, 134.0 m/z; gibberellic acid 3 (GA3): 3.88 min, negative, 34 V, 110 V, 345.13 m/z, 143.1 m/z; indole‐3‐acetic acid (IAA): 4.78 min, negative, 10 V, 90 V, 174.05 m/z, 130.1 m/z; salicylic acid (SA): 4.85 min, negative, 18 V, 90 V, 137.02 m/z, 93.1 m/z; abscisic acid (ABA): 5.15 min, negative, 10 V, 90 V, 263.13 m/z, 153.1 m/z; jasmonic acid (JA): 6.16 min, negative, 10 V, 90 V, 209.12 m/z, 59.1 m/z; gibberellic acid 4 (GA4): 7.06 min, negative, 34 V, 80 V, 331.5 m/z, 213.1 m/z; jasmonic acid‐isoleucine (JA‐Ile): 7.20 min, negative, 22 V, 80 V, 322.20 m/z, 130.1 m/z; methyl jasmonic acid (MeJA): 7.70 min, positive, 10 V, 90 V, 225.15 m/z, 151.1 m/z. Ion chromatograms were extracted and integrated for each phytohormone product ion in MassHunter Qualitative Analysis 10.0 (Agilent Technologies). Peak areas were converted to concentrations (ng/g DW) via external standard calibration curves produced with authentic standards.

### Volatiles

Volatiles were analysed, from 2 g of ground flash frozen tomato fruits, on a GC–MS system with SPME capable autosampler as published in Almeida *et al*. (2021).

Detection of oxo‐β‐ionone was confirmed by another analytical method described in Lewinsohn *et al*. (2001) but using 70 g of frozen fruit tissue. Here, the volatiles were analysed with an Agilent GC 6890 system, coupled to quadrupole mass spectrometer detector 5973 N equipped with Rxi‐5sil MS column (30 m length × 0.25 mm i.d., 0.25 μm film thickness, stationary phase 95% dimethyl–5% diphenyl polysiloxane) and the conditions used were as follows: initial temperature 50 °C for 1 min, followed by a ramp of 5 °C/min to 180 °C, and 10 °C/min up to 280 °C (5 min). The identification of the volatiles was assigned by comparison of their retention indices with those of literature and by comparison of spectral data with standard or with the Nist 98 and QuadLib 2205 GC–MS libraries. Oxo‐β‐ionone was quantified in the keto line (14 ± 2 ng compound/gr tissue/vial) and not detected in the control line.

### Semi‐synthesis and identification of carotenoid‐derived volatiles

Ketolated derivates of β‐ionone and β‐cyclocitral were generated via bromination with DBDMH (1,3‐dibromo‐5,5‐dimethylhydrantoin) and subsequent oxygenation. A 1:1.1:1.5 molar ratio (substrate:DBDMH:NaOH) reaction was stirred at RT (~22 °C) for 5 h while protected from light (Chenyong *et al*., 2015). Products were extracted into methyl tert‐butyl ether (MtBE). Hydroxylated derivatives were generated following reduction of the ketolated derivatives via sodium borohydride. Products were confirmed through EI+ GC–MS via liquid injection. Dried residue (200 μg) of authentic standards (lycopene, β‐carotene, phoenicoxanthin, canthaxanthin, zeaxanthin) in 20 mL headspace was analysed under same condition as tomato fruit samples and manual comparative interpretation of mass spectra undertaken to enhance annotation of carotenoid cleavage products. Identification features used for all compounds analysed are given in Table S15.

### Subchromoplast fractionation on a sucrose gradient

Pericarp, from 10 tomatoes at breaker +5 days ripening stage per tomato line, was cut into 1‐cm^2^ pieces (80–150 g) and stored at 4 °C overnight. Chromoplasts were isolated and subchromoplast compartments were extracted and separated on a sucrose gradient as detailed in Nogueira *et al*. (2013). The identification of the different subchromoplast elements (plastoglobules and membranes) was previously demonstrated by Western blots in Nogueira *et al*. (2013).

### Determination of in vivo chlorophyll fluorescence

In vivo chlorophyll fluorescence was measured using a pocket PEA chlorophyll fluorimeter (Hansatech Instruments, King's Lynn, UK) as described in Enfissi *et al*. (2019). Per tomato line, five leaves were analysed for each six biological replicates.

### Transmission electron microscopy

Per tomato line, three tomato fruits, from three different biological replicates, were cut into 3‐mm cubes using a razor blade on a tile, and they were placed in a vial containing (~3 mL) room temperature fixative (2.5% glutaraldehyde in 0.01 M phosphate‐buffered saline (PBS) at pH 7.2) and placed under vacuum for 20 s to aid infiltration. Samples were placed on a rotator for ~5 h before going in the fridge (4 °C) overnight. Tissue was washed in PBS 2 × 20 min and then post‐fixed in 1% osmium tetroxide in PBS for 1 h at RT. Tissue was then dehydrated in increasing concentrations of acetone as follows: 10%, 30%, 50%, 70%, and 90% and 3 × 100% (minimum 1 h for each wash). Tissue was then infiltrated in increasing concentrations of resin diluted with acetone and placed under vacuum for 20 s as follows: 30%, 70% and 2 × 100% Spurr resin (minimum 1.5 h for each wash). Spurr medium ERL4221D (TAAB Laboratories Equipment Ltd.) was used and freshly mixed on the day. Tissue pieces were then placed in labelled capsules containing fresh resin and polymerized in the oven (60 °C) for 16 h. Polymerized blocks were sectioned at 70 nm on a Leica Ultracut UC7, sections were collected on 200 mesh copper grids coated with carbon–formvar. Sections were counterstained with 2.5% uranyl acetate in distilled water for 20 min, washed with distilled water, stained with Reynolds lead citrate for 3 min and then washed with distilled water again. Regions of interest were viewed in a JEOL JEM‐2100 Plus Transmission Electron Microscope with an accelerating voltage of 200 kV. Images were recorded with a Gatan OneView IS camera. The images shown in Figure 2 are representative of three biological replicates for each line, from which 12–20 regions of interest were imaged per biological replicate. Plastid measurements were determined using ImageJ software. In particular, the colour intensity of the plastoglobules (grey ratio) was calculating by measuring the colour intensity within several plastoglobules at three locations within the plastoglobules of an image and normalizing this value to the colour intensity of the background (stroma) of this image (9 positions per image).

### RNA‐seq

RNeasy Plant Mini kit (Qiagen) was used to extract total RNA from 39 and 49 dpa tomato fruit following manufacturer's instructions from three tomatoes per biological replicate. Three biological replicates per line were studied. Single‐read mRNA sequencing (1x100bp) was carried out with HiSeq2500 (Illumina, San Diego, CA). TruSeq Stranded mRNA Sample Prep kit (Illumina, San Diego, CA) has been used for library preparation following the manufacturer's instructions, starting with 1–2 μg of good quality RNA (R.I.N. >7) as input. The poly‐A mRNA was fragmented 3 min at 94 °C, and every purification step has been performed by using 1X Agencourt AMPure XP beads. Both RNA samples and final libraries were quantified by using the Qubit 2.0 Fluorometer (Invitrogen, Carlsbad, CA) and quality tested by Agilent 2100 Bioanalyzer RNA Nano assay (Agilent technologies, Santa Clara, CA). Libraries were then processed with Illumina cBot for cluster generation on the flow cell, following the manufacturer's instructions and sequenced on single‐end mode at the multiplexing level requested on HiSeq2500. The CASAVA 1.8.2 version of the Illumina pipeline was used to processed raw data for both format conversion and de‐multiplexing. RNA‐Seq standard bioinformatics analysis were performed (Trapnell *et al*., 2012), including base calling and demultiplexing, trimming (with ERNE1 and Cutadapt2), alignments with TopHat2, transcripts count (Cufflinks3), quality control using RSeqQC4 package and pairwise differential expression analysis (Cuffdiff5). FPKM normalization was used in this study. Differentially expressed genes (DEGs) were considered significant when FDR ≤0.05. Mapping of reads to the *Solanum lycopersicum* SL3 (NCBI_SL3.0_protein) genome and Gene Ontology annotation was achieved with OmicsBox software (Bioinformatics Made Easy, BioBam Bioinformatics, March 3, 2019, https://www.biobam.com/omicsbox, previously Blast2GO, Götz *et al*., 2008) as well as the Gene Ontology enrichment analyses (Fisher's exact test, FDR ≤0.05) comparing two sets of DEGs. The Gene Ontology enrichment analysis of a single DEG set was completed on g:Profiler web server. The percentage of mapped reads obtained were as follows: for the DEGs of the ripe and mature green stage comparisons, respectively, keto/control: 82% and 78%, β‐carotene/control: 80% and 76%, and keto/β‐carotene: 83% at both stages, and for the DEGs of the ripe/mature green comparisons, 81% of the reads were mapped for each tomato line.

### Correlation network

Computations of the correlations were conducted in XLSTAT. Pearson correlation analysis was employed to compute all pairwise correlations between log2 of fold change metabolite levels and log2 of fold change expression levels for significantly different metabolites and DEGs of the ketocarotenoid line compared to the control (*P*‐value <0.5). Cytoscape was used to generate graphical output of network.

### Statistical analysis

Analyses were performed on datasets comprising three to six biological replicates and at least three technical replicates. N represented the number of biological replicates, that is the number of plants studied per line. Analyses were made upon three pooled fruits per biological replicates. The technical replicates consisted in multiple extractions of each biological replicate. Absolute quantification was achieved by using dose–response curves of authentic standards. When standards were not commercially available, relative quantification was performed. Statistical analyses were carried out using SPPS 25 software (IBM) and XLSTAT (Addinsoft). Comparison between two and more than two lines was subjected to independent samples t‐test and one‐way ANOVA statistical analysis, respectively. Equality of variances was tested with a Levene's test. If the homogeneity of variance was assumed, the *P*‐value selected was, for t‐test, the one for ‘equal variance assumed’ and, for ANOVA, the one for the Tukey post hoc test. However, if the assumption of the homogeneity of variance was violated, then the *P*‐value corresponding to ‘equal variance not assumed’ was chosen for the t‐test and, for the ANOVA, the one corresponding to the Games‐Howell post hoc. Means and standard deviation were calculated in Excel as well as Pearson correlation (*P*‐corr, *P*‐value). SIMCA 17 (Sartorius) was used to carry out and display clusters derived from univariate scaling applied data for principal component analysis (PCA) and to perform ‘bottom‐up’ hierarchal clustering. *P*‐value of statistical tests performed is described in Table S13 if not already presented as supplemental data. Randomization technique was used whenever possible, such as running GC–MS and LC–MS samples in a random order. Microsoft Excel software was used to create randomized sequences.

## Author contributions

M.N., P.D.F. and E.M.A.E. designed the study and its conceptualization; M.N., E.J.P., G.N.M. and E.V. performed the research; M.N. analysed the data; and M.N., P.D.F. and E.M.A.E wrote the manuscript with the input from all authors. PDF and E.M.A.E were responsible for the funding.

## Conflict of interest

The authors declare no conflict of interest.

## Supporting information


**Figure S1** Differential expression of the carotenogenic genes in the ketocarotenoid MG and ripe fruits compared to their respective controls.

**Figure S2** Ketocarotenoid chromatographic profiles of the ketocarotenoid fruit at mature green and ripe stages.

**Figure S3** Chlorophyll content in fruit at different developmental and ripening stages.

**Figure S4** Quantification of carotenoids and chlorophylls in leaves and measurement of *in vivo* chlorophyll fluorescence (Fv/Fm).

**Figure S5** Carotenoid quantification of the fruit chromoplast fractions.

**Figure S6** Principal component analysis of all metabolites quantified in ripe fruit including or excluding the carotenoid data and metabolic hierarchical clustering of the tomato lines.

**Figure S7** Biochemical pathways displaying the fold change metabolite levels in ripe and mature green fruit of the β‐carotene line compared to the control.

**Figure S8** Principal component analysis of all metabolites quantified in mature green fruit including or excluding the carotenoid data.

**Figure S9** Differentially expressed genes and enrichment analysis of the keto/β‐carotene comparison.

**Figure S10** Hierarchical clustering of the control, β‐carotene and ketocarotenoid line RNA‐seq data.

**Figure S11** Gene Ontology enrichment analysis of the keto/control and β‐carotene/control comparison at MG.

**Figure S12** Transcriptional changes in the metabolic pathways of the ripe fruit of the β‐carotene line compared to the control.

**Table S1** Carotenoid quantification in fruit over five ripening stages.

**Table S2** Electron microscopy related measurements of mature green and ripe tomato fruit electron micrographs.

**Table S3** Carotenoid quantification of the fruit chromoplast fractions.

**Table S4** Metabolite content in the ripe fruit.

**Table S5** Metabolite content in the mature green (MG) fruit.

**Table S6** Gene Ontology enrichment analysis lists.

**Table S7** List of RNA‐seq DEGs used to create pathway visualization displays.

**Table S8** Expression data of plastidial redox regulatory genes.

**Table S9** Gene‐metabolite correlation matrix of the ripe keto line / control comparison.

**Table S10** Gene enrichment analysis of the down‐regulated Ripe/MG DEGs of the keto line versus the control line.

**Table S11** Gene enrichment analysis of the up‐regulated DEGs of the keto/control versus β‐caro/control comparison in ripe fruit.

**Table S12** Gene enrichment analysis of the common DEGs from the keto/control and Keto/β‐caro sets in ripe fruit.

**Table S13** Statistical analysis.

**Table S14** Chromatographic details of compounds analysed by UPLC.

**Table S15** Metabolite identification features.

**Data S1** Supporting Information.

## Data Availability

Raw RNA‐seq data have been deposited at the European Nucleotide Archive (Project accession PRJEB57363) and are publicly available as of the date of publication.
