# Supplementary material for: Ketocarotenoid production in tomato triggers metabolic reprogramming and cellular adaptation: The quest for homeostasis
Source: Plant Biotechnol J. 2023 Nov 30;22(2):427–44. doi: 10.1111/pbi.14196 (PMC10826984; doi:10.1111/pbi.14196)
Supplement: Supplementary file 1 — Figure S1 Differential expression of the carotenogenic genes in the ketocarotenoid MG and ripe fruits compared to their respective controls. [file PBI-22-427-s013.pptx]

## Slide 1
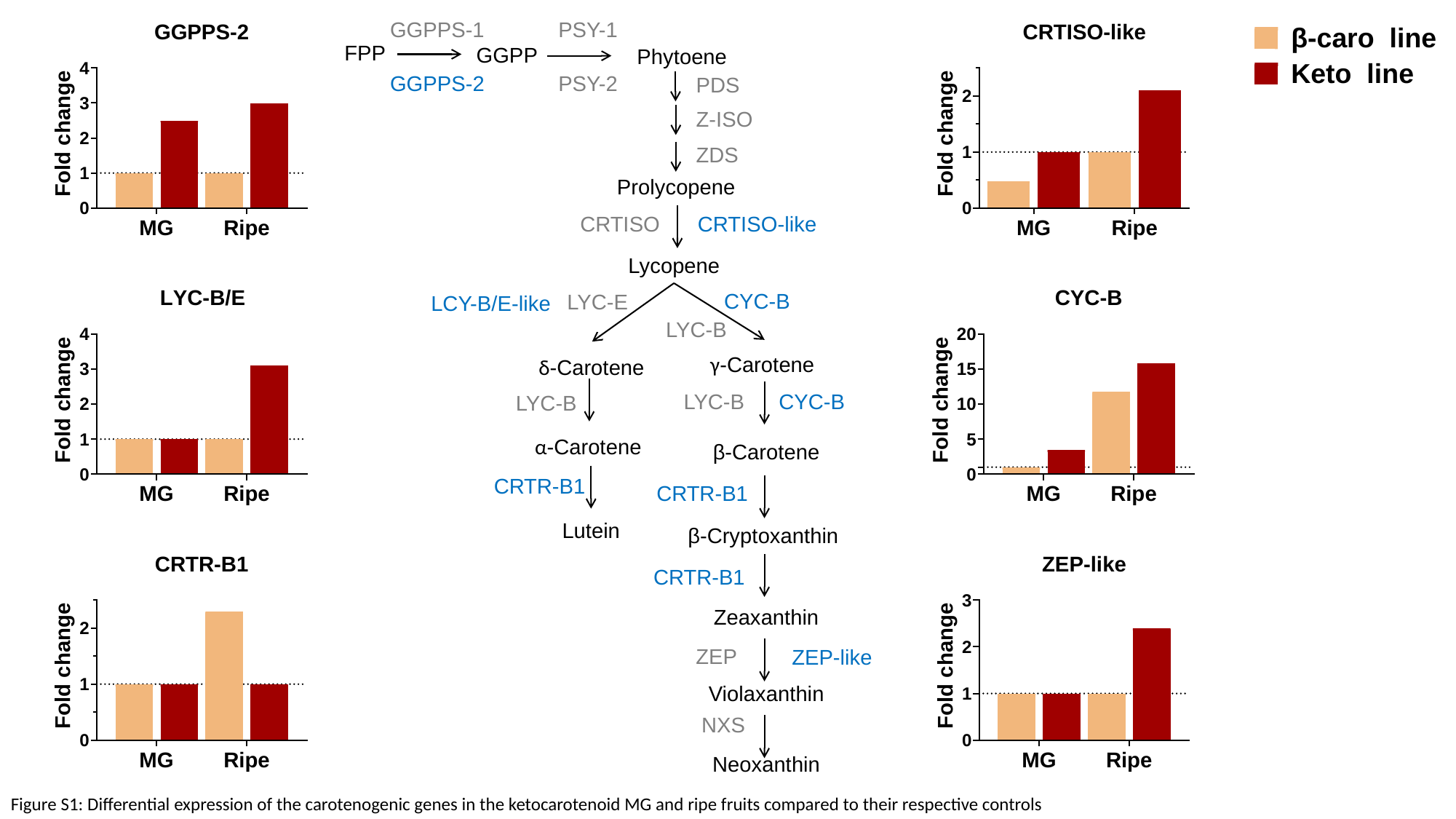

GGPPS-1
PSY-1
FPP
GGPP
Phytoene
GGPPS-2
PSY-2
PDS
Z-ISO
ZDS
Prolycopene
CRTISO-like
CRTISO
Lycopene
CYC-B
LYC-E
LCY-B/E-like
LYC-B
γ-Carotene
δ-Carotene
LYC-B
CYC-B
LYC-B
α-Carotene
β-Carotene
CRTR-B1
CRTR-B1
Lutein
β-Cryptoxanthin
CRTR-B1
Zeaxanthin
ZEP
ZEP-like
Violaxanthin
NXS
Neoxanthin
Figure S1: Differential expression of the carotenogenic genes in the ketocarotenoid MG and ripe fruits compared to their respective controls
