# Supplementary material for: Ketocarotenoid production in tomato triggers metabolic reprogramming and cellular adaptation: The quest for homeostasis
Source: Plant Biotechnol J. 2023 Nov 30;22(2):427–44. doi: 10.1111/pbi.14196 (PMC10826984; doi:10.1111/pbi.14196)
Supplement: Supplementary file 2 — Figure S2 Ketocarotenoid chromatographic profiles of the ketocarotenoid fruit at mature green and ripe stages. [file PBI-22-427-s021.pptx]

## Slide 1
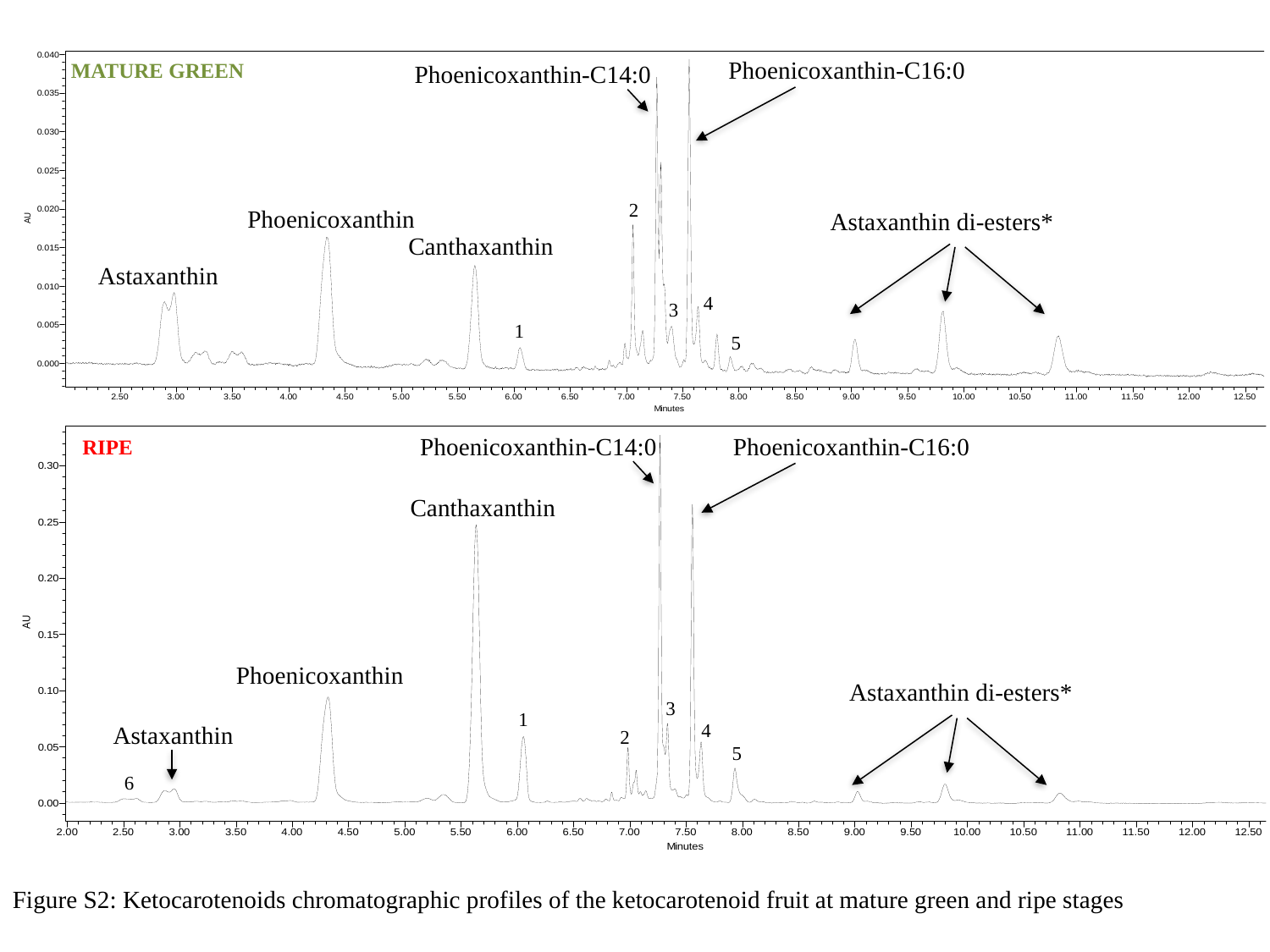

Phoenicoxanthin-C16:0
MATURE GREEN
Phoenicoxanthin-C14:0
2
Phoenicoxanthin
Astaxanthin di-esters*
Canthaxanthin
Astaxanthin
4
3
1
5
Phoenicoxanthin-C14:0
Phoenicoxanthin-C16:0
RIPE
Canthaxanthin
Phoenicoxanthin
Astaxanthin di-esters*
3
1
4
Astaxanthin
2
5
6
Figure S2: Ketocarotenoids chromatographic profiles of the ketocarotenoid fruit at mature green and ripe stages
