# Supplementary material for: Ketocarotenoid production in tomato triggers metabolic reprogramming and cellular adaptation: The quest for homeostasis
Source: Plant Biotechnol J. 2023 Nov 30;22(2):427–44. doi: 10.1111/pbi.14196 (PMC10826984; doi:10.1111/pbi.14196)
Supplement: Supplementary file 3 — Figure S3 Chlorophyll content in fruit at different developmental and ripening stages. [file PBI-22-427-s023.pptx]

## Slide 1
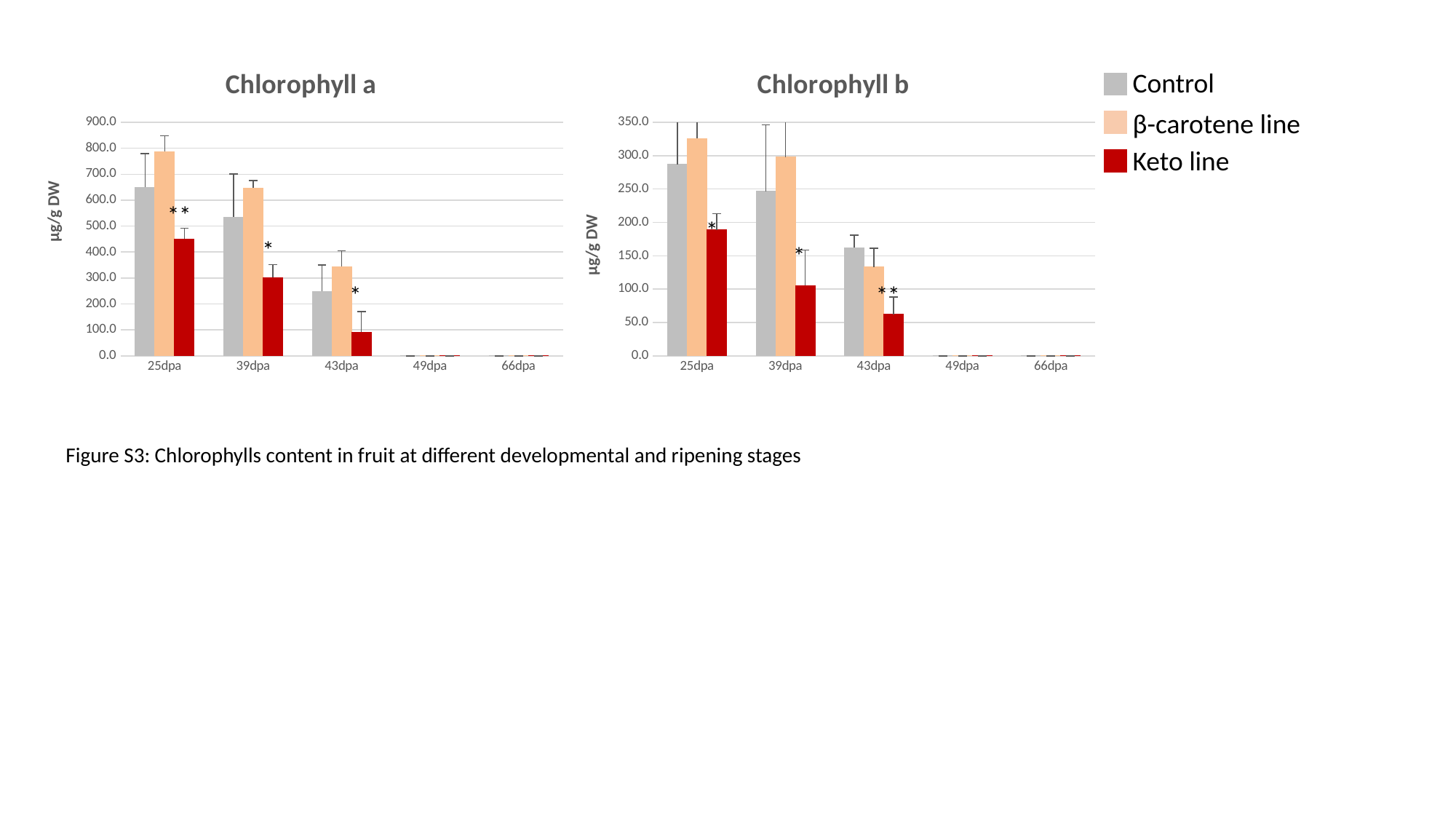

### Chart: Chlorophyll a
| Category | Control | β-caro. line | Keto. line |
|---|---|---|---|
| 25dpa | 649.5898730607745 | 787.3193652380014 | 449.8303944626828 |
| 39dpa | 534.7391692671969 | 647.3640442925786 | 300.99530720224305 |
| 43dpa | 248.9028141137137 | 343.8422329632069 | 91.37278455341476 |
| 49dpa | 0.0 | 0.0 | 0.0 |
| 66dpa | 0.0 | 0.0 | 0.0 |
### Chart: Chlorophyll b
| Category | Control | β-caro. line | Keto. line |
|---|---|---|---|
| 25dpa | 287.354824276736 | 325.9135234744867 | 188.92958031258456 |
| 39dpa | 246.68312402648667 | 298.14044994861797 | 105.57492875616776 |
| 43dpa | 161.8638632817879 | 133.35589434614496 | 62.079070711158145 |
| 49dpa | 0.0 | 0.0 | 0.0 |
| 66dpa | 0.0 | 0.0 | 0.0 |Control
β-carotene line
Keto line
**
*
*
*
*
**
Figure S3: Chlorophylls content in fruit at different developmental and ripening stages
