# Supplementary figures and images for: Ketocarotenoid production in tomato triggers metabolic reprogramming and cellular adaptation: The quest for homeostasis
Source: Plant Biotechnol J. 2023 Nov 30;22(2):427–44. doi: 10.1111/pbi.14196 (PMC10826984; doi:10.1111/pbi.14196)

## Slide 1
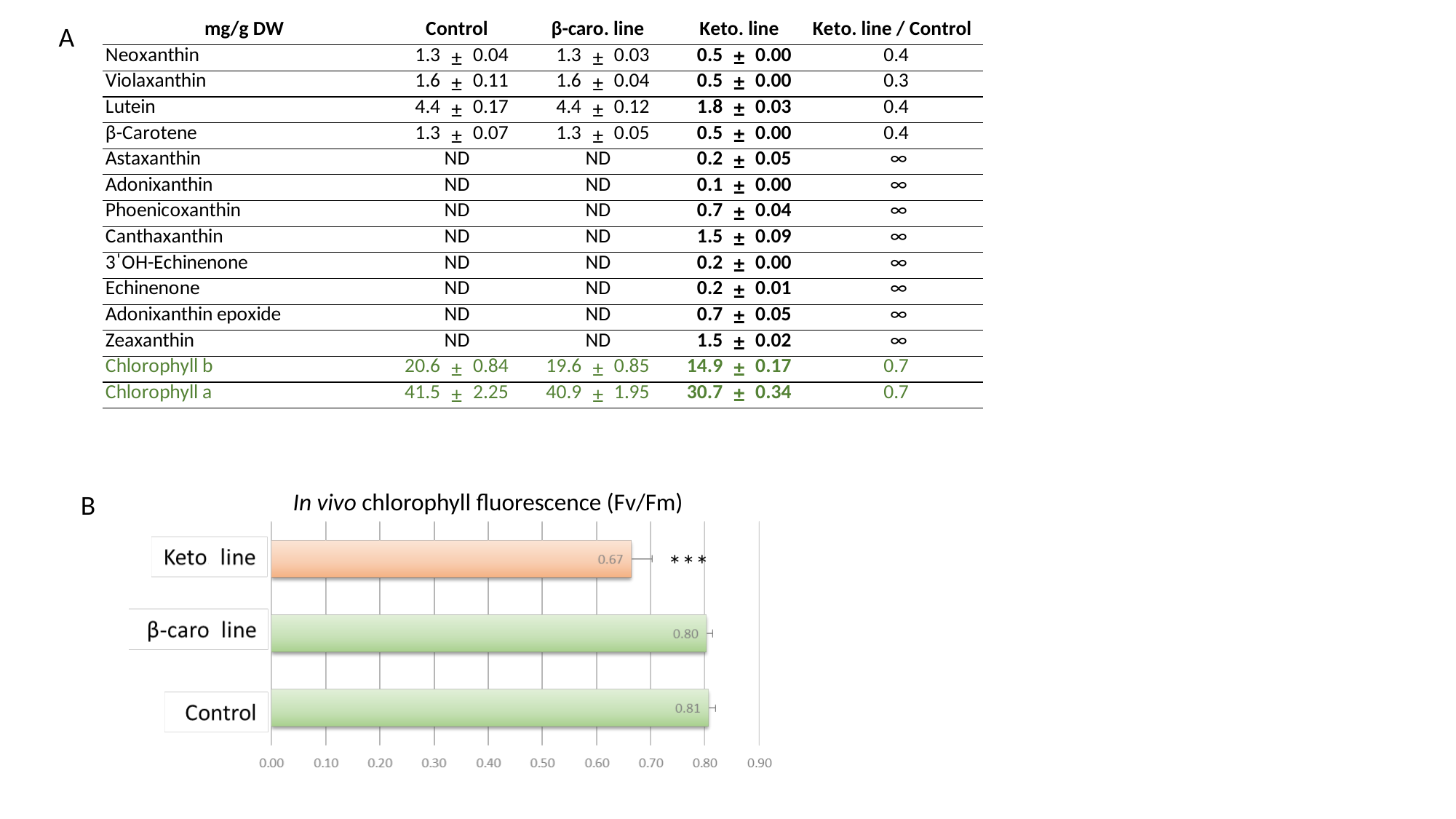

A
In vivo chlorophyll fluorescence (Fv/Fm)
***
B

Supplement: Supplementary file 4 — Figure S4 Quantification of carotenoids and chlorophylls in leaves and measurement of in vivo chlorophyll fluorescence (Fv/Fm). [file PBI-22-427-s009.pptx]

## Slide 1
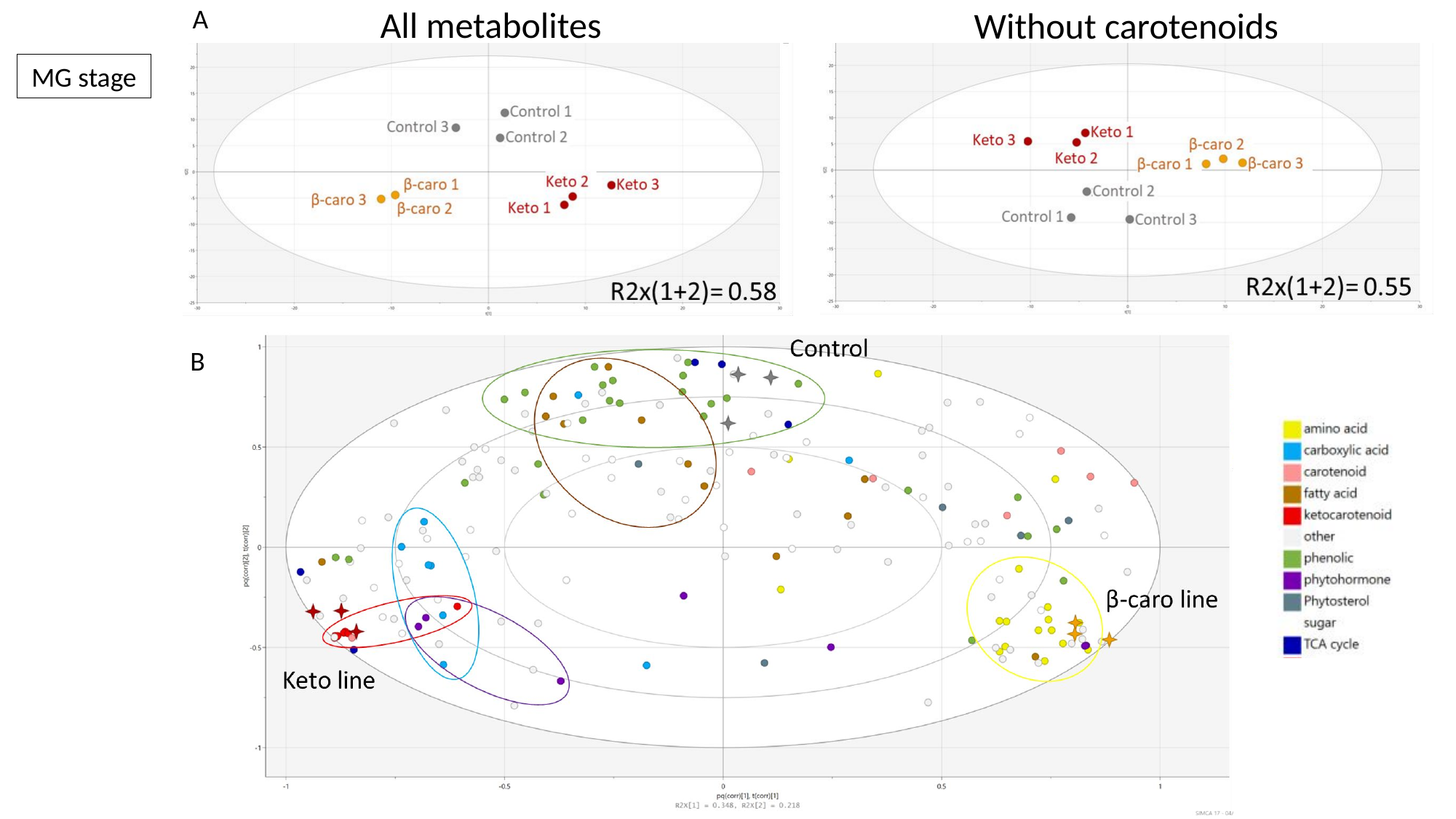

A
All metabolites
Without carotenoids
MG stage
B

Supplement: Supplementary file 8 — Figure S8 Principal component analysis of all metabolites quantified in mature green fruit including or excluding the carotenoid data. [file PBI-22-427-s005.pptx]

## Slide 1
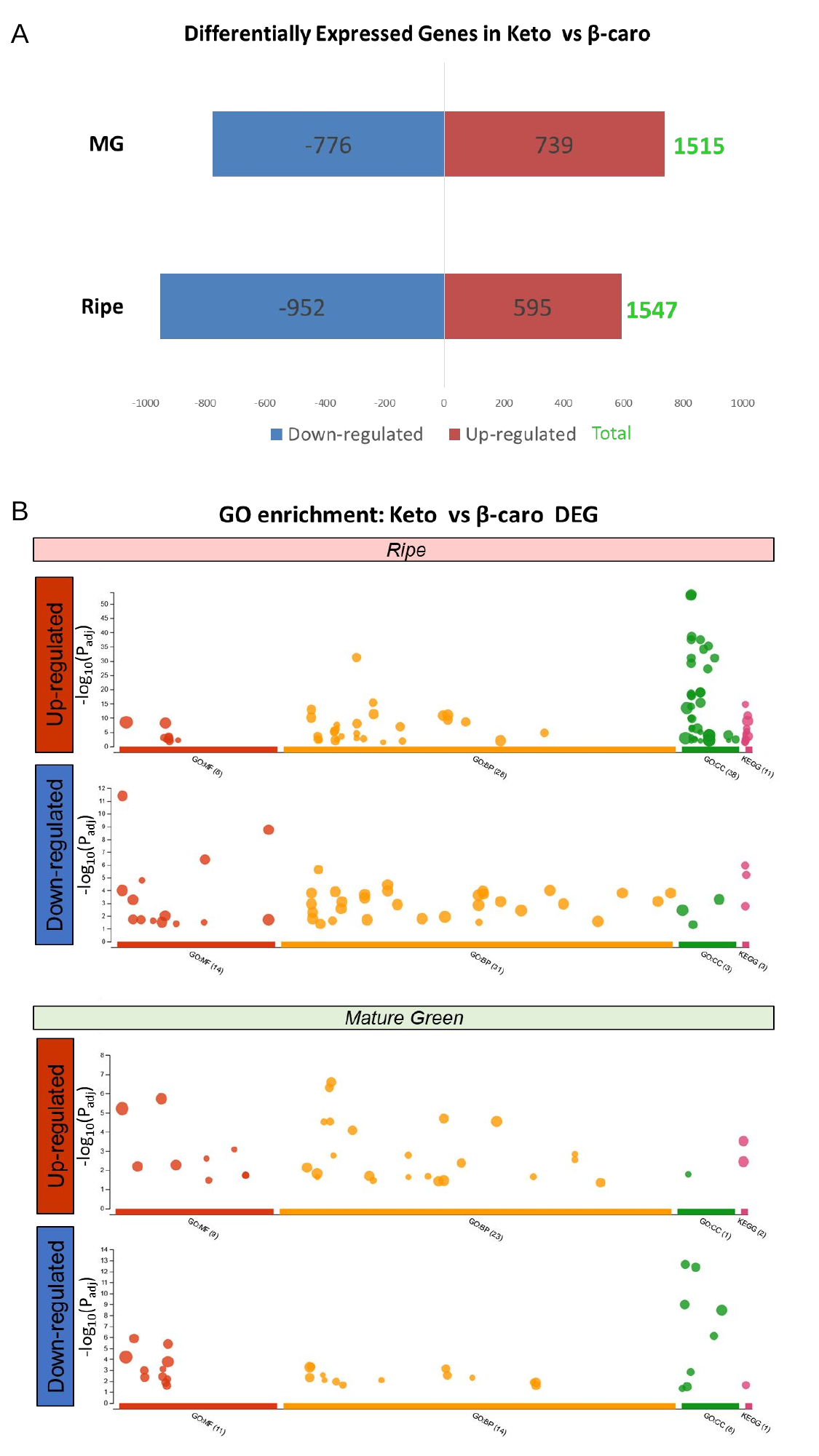

A
B

Supplement: Supplementary file 9 — Figure S9 Differentially expressed genes and enrichment analysis of the keto/β‐carotene comparison. [file PBI-22-427-s017.pptx]

## Slide 1
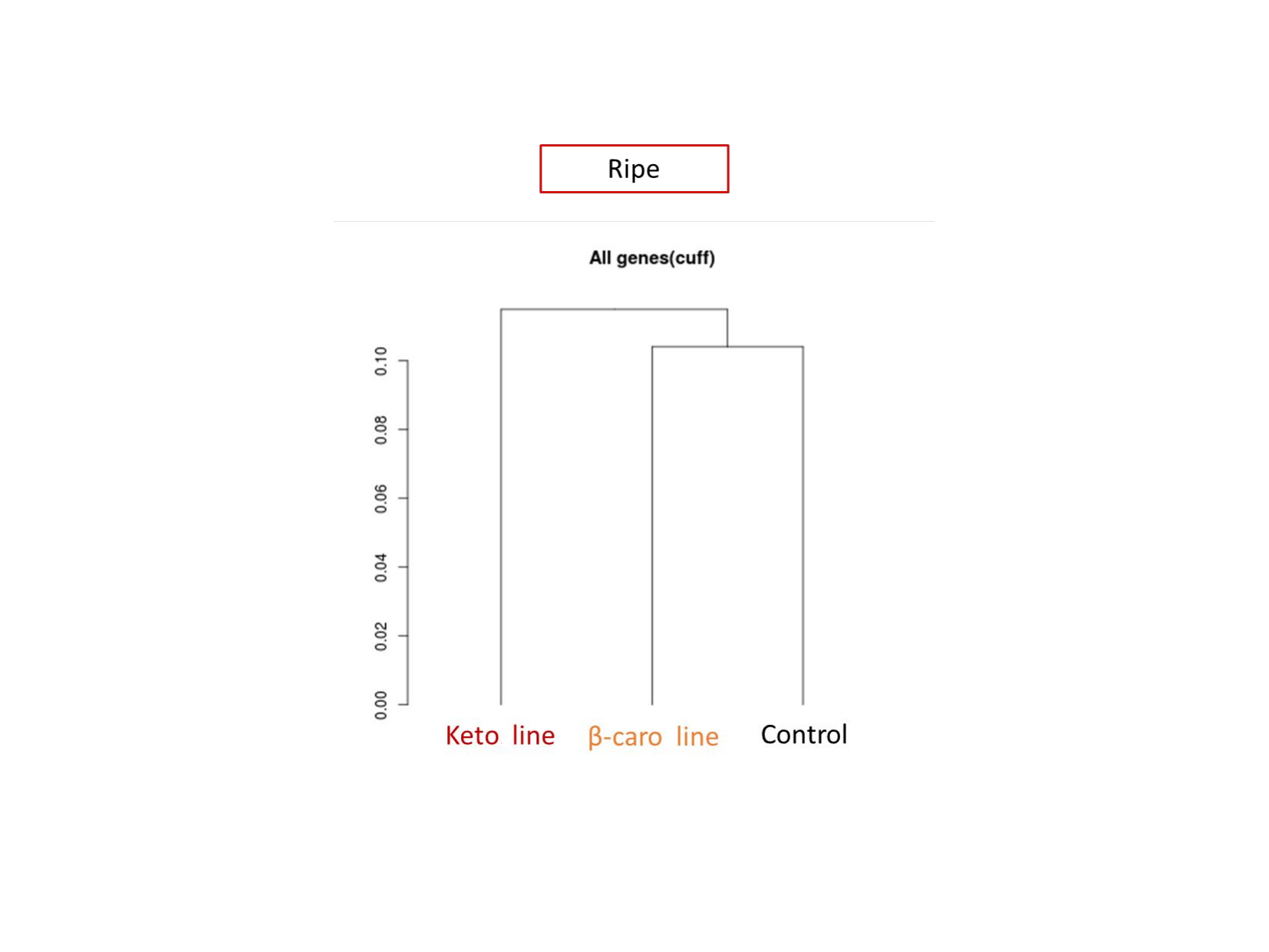

Supplement: Supplementary file 10 — Figure S10 Hierarchical clustering of the control, β‐carotene and ketocarotenoid line RNA‐seq data. [file PBI-22-427-s020.pptx]
