# Supplementary material for: Ketocarotenoid production in tomato triggers metabolic reprogramming and cellular adaptation: The quest for homeostasis
Source: Plant Biotechnol J. 2023 Nov 30;22(2):427–44. doi: 10.1111/pbi.14196 (PMC10826984; doi:10.1111/pbi.14196)
Supplement: Supplementary file 5 — Figure S5 Carotenoid quantification of the fruit chromoplast fractions. [file PBI-22-427-s011.pptx]

## Slide 1
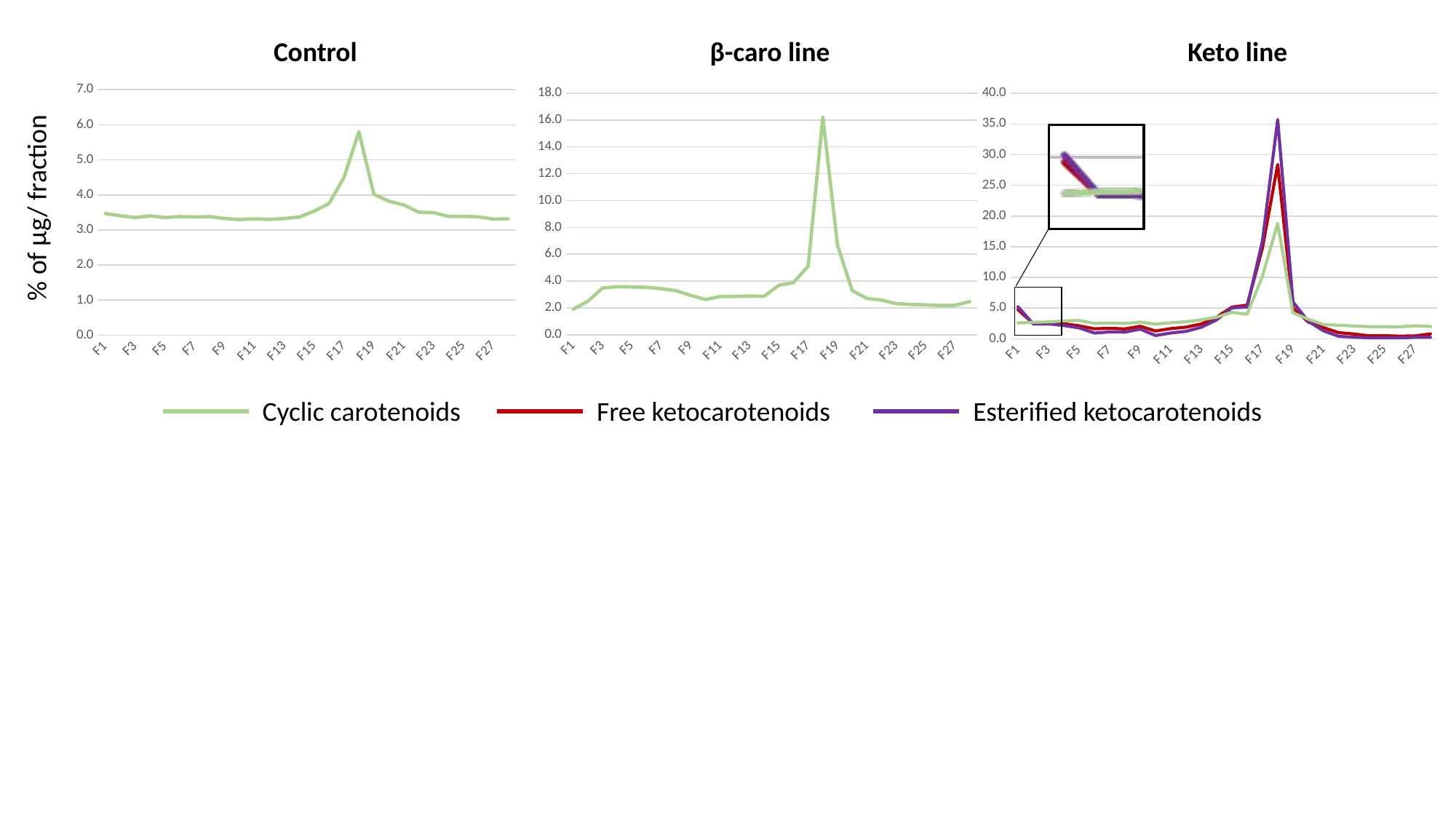

Control
β-caro line
Keto line
### Chart
| Category | Cyclic carotenoids |
|---|---|
| F1 | 3.4690167860705916 |
| F2 | 3.40503769592166 |
| F3 | 3.355913341260203 |
| F4 | 3.4013832079026343 |
| F5 | 3.3570952403599743 |
| F6 | 3.3839922086941194 |
| F7 | 3.369028904952016 |
| F8 | 3.3819922359225427 |
| F9 | 3.3280889261263553 |
| F10 | 3.2988326670507764 |
| F11 | 3.3188772105120923 |
| F12 | 3.301880797075121 |
| F13 | 3.32767230127997 |
| F14 | 3.3696739715595574 |
| F15 | 3.536527148476494 |
| F16 | 3.7556447849781347 |
| F17 | 4.500513608081866 |
| F18 | 5.800045823879904 |
| F19 | 4.019539270732097 |
| F20 | 3.819566710349507 |
| F21 | 3.715683778751791 |
| F22 | 3.5086083293921315 |
| F23 | 3.4945138376431895 |
| F24 | 3.388253917556617 |
| F25 | 3.390111228757944 |
| F26 | 3.375303205175745 |
| F27 | 3.3114930656232957 |
| F28 | 3.3157097959136865 |
### Chart
| Category | Cyclic carotenoids |
|---|---|
| F1 | 1.900049875761645 |
| F2 | 2.505460753462894 |
| F3 | 3.4805649267017706 |
| F4 | 3.577984605088193 |
| F5 | 3.557226923305036 |
| F6 | 3.5308002598932475 |
| F7 | 3.4326881774040974 |
| F8 | 3.2743461974245793 |
| F9 | 2.9313660372820025 |
| F10 | 2.626205266259508 |
| F11 | 2.8507263168365085 |
| F12 | 2.850381798564624 |
| F13 | 2.8814487933409403 |
| F14 | 2.863186592067438 |
| F15 | 3.684887883995214 |
| F16 | 3.880594864167597 |
| F17 | 5.109797394920165 |
| F18 | 16.207394899417817 |
| F19 | 6.659153524423916 |
| F20 | 3.2879107443218873 |
| F21 | 2.7022704383015794 |
| F22 | 2.577648188355765 |
| F23 | 2.3142317365977108 |
| F24 | 2.2558619449832324 |
| F25 | 2.2256511834165273 |
| F26 | 2.1765038479425063 |
| F27 | 2.188857031006795 |
| F28 | 2.466799794756808 |
### Chart
| Category | Free ketocarotenoids | Ketocartenoid esters | Carotenoids |
|---|---|---|---|
| F1 | 4.740004673268766 | 5.219281393978733 | 2.5840919242311653 |
| F2 | 2.482288040560878 | 2.408675646359131 | 2.6955403289019326 |
| F3 | 2.5584106386317305 | 2.4266725189670453 | 2.7392805885648017 |
| F4 | 2.454336961103713 | 2.1675687027456316 | 2.918940725809759 |
| F5 | 2.116544731716164 | 1.782294560945763 | 2.9855882995380862 |
| F6 | 1.6270361411269063 | 0.9353170624561093 | 2.508331946304233 |
| F7 | 1.7118333066964235 | 1.115106220501459 | 2.573893885964415 |
| F8 | 1.613547360350278 | 1.0885280264691328 | 2.4990044378213607 |
| F9 | 2.043522422050837 | 1.5758661001705332 | 2.7171316109774195 |
| F10 | 1.270137700501079 | 0.5387745557459472 | 2.3840377722817903 |
| F11 | 1.6655910086934766 | 0.9436192388812176 | 2.6091478530932704 |
| F12 | 1.897454561991239 | 1.2182774929790132 | 2.7836730264207685 |
| F13 | 2.418749125586086 | 1.889109296878938 | 3.083235080493872 |
| F14 | 3.5222193894133524 | 3.080808875521752 | 3.525641928633808 |
| F15 | 5.157742114677962 | 5.0403342925669286 | 4.304590107804132 |
| F16 | 5.490123567470877 | 5.166800712262314 | 3.9921729823279946 |
| F17 | 14.785513954334828 | 15.929428589955538 | 10.18781139415806 |
| F18 | 28.40230616106908 | 35.71876910317983 | 18.81118611468199 |
| F19 | 5.012930844070811 | 5.990936932420453 | 4.235244494607857 |
| F20 | 2.7354795664653837 | 2.852731593528796 | 3.1904216037361257 |
| F21 | 1.8134638019876217 | 1.2888490308486527 | 2.329470900144749 |
| F22 | 0.9996098505064728 | 0.40444140363267383 | 2.2103907539585568 |
| F23 | 0.7822500339749677 | 0.268042873883646 | 2.093346928747235 |
| F24 | 0.4897670976516194 | 0.15949601020976376 | 1.9783825303069813 |
| F25 | 0.5003780031210919 | 0.16102111243390352 | 1.974580857857129 |
| F26 | 0.43865469452687234 | 0.15061264319019285 | 1.9607218543637348 |
| F27 | 0.4714732264657581 | 0.23900336443384992 | 2.1179803032925846 |
| F28 | 0.7986310219857146 | 0.23963264485303917 | 2.0061597649761955 |
% of µg/ fraction
Cyclic carotenoids
Free ketocarotenoids
Esterified ketocarotenoids
