# Supplementary material for: Ketocarotenoid production in tomato triggers metabolic reprogramming and cellular adaptation: The quest for homeostasis
Source: Plant Biotechnol J. 2023 Nov 30;22(2):427–44. doi: 10.1111/pbi.14196 (PMC10826984; doi:10.1111/pbi.14196)
Supplement: Supplementary file 6 — Figure S6 Principal component analysis of all metabolites quantified in ripe fruit including or excluding the carotenoid data and metabolic hierarchical clustering of the tomato lines. [file PBI-22-427-s027.pptx]

## Slide 1
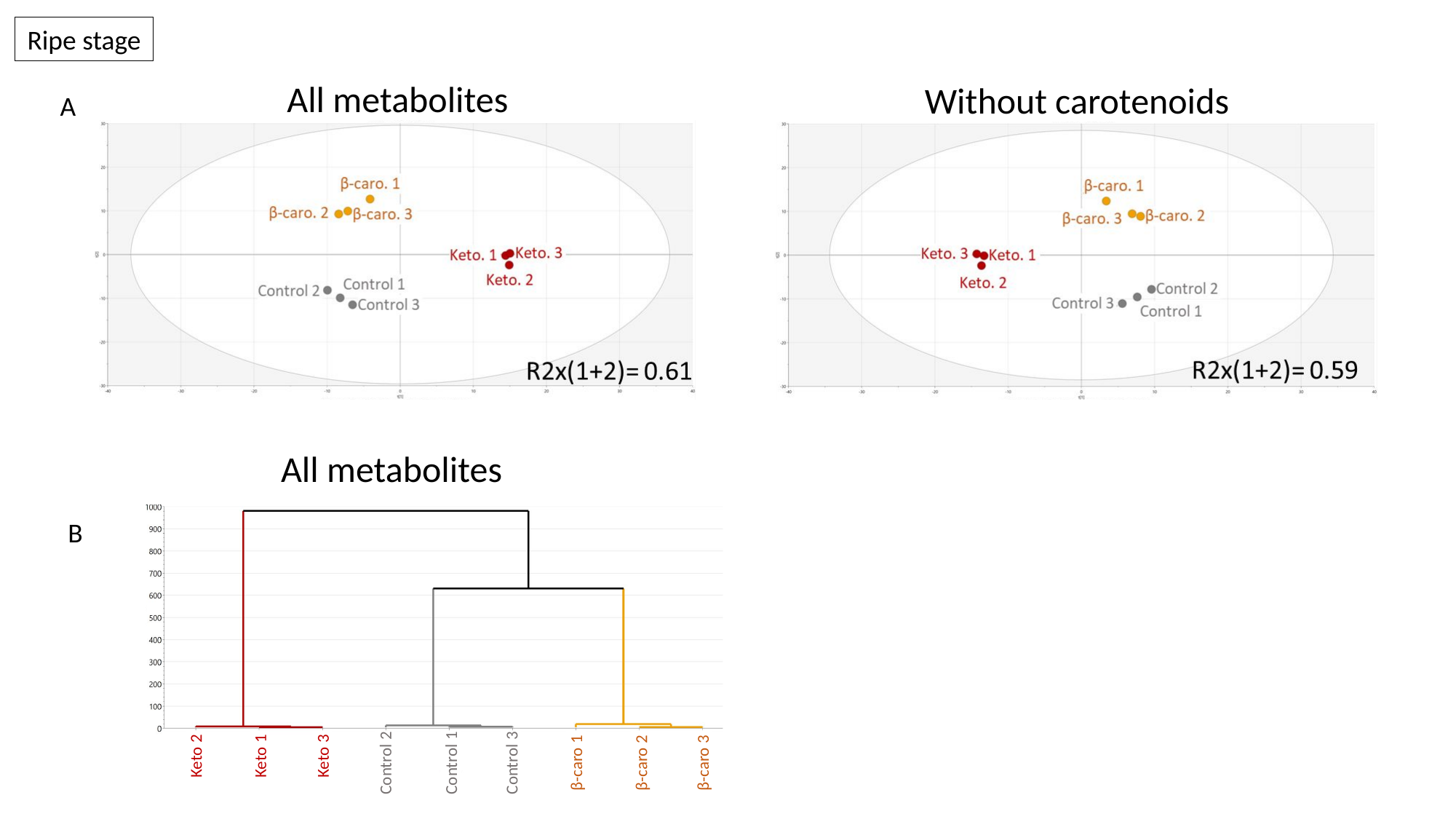

Ripe stage
All metabolites
Without carotenoids
A
All metabolites
B
Keto 2
Keto 1
Keto 3
Control 2
Control 1
Control 3
β-caro 1
β-caro 2
β-caro 3
