# Supplementary material for: Ketocarotenoid production in tomato triggers metabolic reprogramming and cellular adaptation: The quest for homeostasis
Source: Plant Biotechnol J. 2023 Nov 30;22(2):427–44. doi: 10.1111/pbi.14196 (PMC10826984; doi:10.1111/pbi.14196)
Supplement: Supplementary file 7 — Figure S7 Biochemical pathways displaying the fold change metabolite levels in ripe and mature green fruit of the β‐carotene line compared to the control. [file PBI-22-427-s025.pptx]

## Slide 1
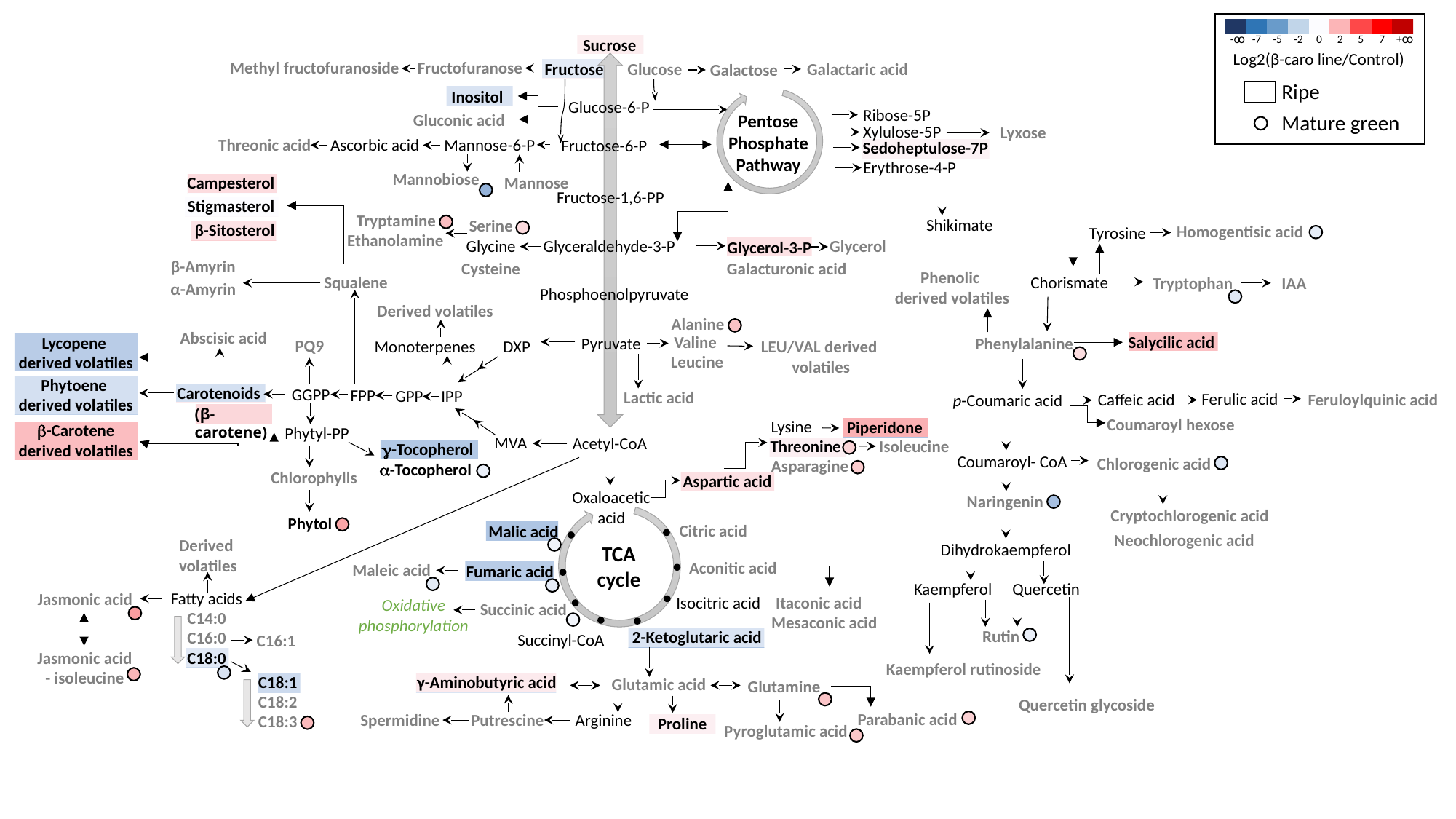

Sucrose
Log2(β-caro line/Control)
Methyl fructofuranoside
Fructofuranose
Fructose + Glucose
Galactaric acid
Galactose
Ripe
Inositol
Glucose-6-P
Ribose-5P
Gluconic acid
Mature green
Pentose Phosphate Pathway
Xylulose-5P
Lyxose
Ascorbic acid
Threonic acid
Mannose-6-P
Fructose-6-P
Sedoheptulose-7P
Erythrose-4-P
Mannobiose
Campesterol
Mannose
Fructose-1,6-PP
Stigmasterol
Tryptamine
Shikimate
Serine
β-Sitosterol
Homogentisic acid
Tyrosine
Ethanolamine
Glycerol
Glyceraldehyde-3-P
Glycine
Glycerol-3-P
β-Amyrin
Cysteine
Galacturonic acid
Phenolic
derived volatiles
Chorismate
Squalene
Tryptophan
IAA
α-Amyrin
Phosphoenolpyruvate
Derived volatiles
Alanine
Abscisic acid
Salycilic acid
Valine
Lycopene
derived volatiles
Phenylalanine
Pyruvate
PQ9
DXP
Monoterpenes
LEU/VAL derived
 volatiles
Leucine
Phytoene
derived volatiles
Carotenoids
GGPP
FPP
GPP
IPP
Lactic acid
Ferulic acid
Feruloylquinic acid
Caffeic acid
p-Coumaric acid
(β-carotene)
Coumaroyl hexose
Lysine
Piperidone
β-Carotene derived volatiles
Phytyl-PP
MVA
Acetyl-CoA
Isoleucine
Threonine
γ-Tocopherol
Coumaroyl- CoA
Chlorogenic acid
Asparagine
α-Tocopherol
Chlorophylls
Aspartic acid
Oxaloacetic acid
Naringenin
Cryptochlorogenic acid
Phytol
Citric acid
Malic acid
Neochlorogenic acid
Derived
 volatiles
Dihydrokaempferol
TCA cycle
Aconitic acid
Maleic acid
Fumaric acid
Kaempferol
Quercetin
Fatty acids
C14:0
C16:0
C18:0
Jasmonic acid
Itaconic acid
Isocitric acid
Oxidative phosphorylation
Succinic acid
Mesaconic acid
Rutin
2-Ketoglutaric acid
Succinyl-CoA
C16:1
Jasmonic acid - isoleucine
Kaempferol rutinoside
C18:1
C18:2
C18:3
γ-Aminobutyric acid
Glutamic acid
Glutamine
Quercetin glycoside
Parabanic acid
Spermidine
Arginine
Putrescine
Proline
Pyroglutamic acid
