# Supplementary material for: Ketocarotenoid production in tomato triggers metabolic reprogramming and cellular adaptation: The quest for homeostasis
Source: Plant Biotechnol J. 2023 Nov 30;22(2):427–44. doi: 10.1111/pbi.14196 (PMC10826984; doi:10.1111/pbi.14196)
Supplement: Supplementary file 11 — Figure S11 Gene Ontology enrichment analysis of the keto/control and β‐carotene/control comparison at MG. [file PBI-22-427-s024.pptx]

## Slide 1
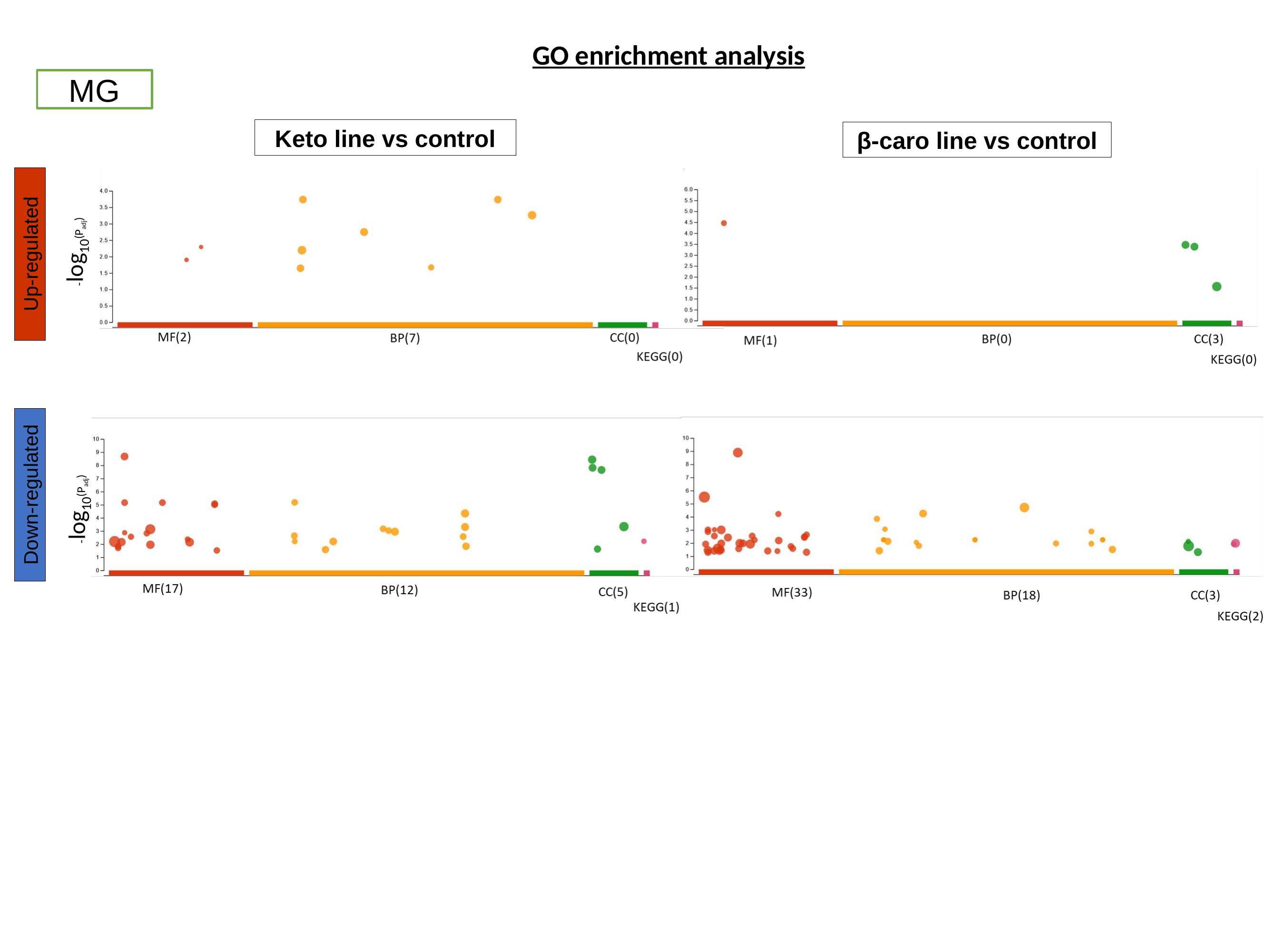

GO enrichment analysis
MG
Keto line vs control
β-caro line vs control
-log10(Padj)
Up-regulated
Down-regulated
-log10(Padj)
