# Supplementary material for: Ketocarotenoid production in tomato triggers metabolic reprogramming and cellular adaptation: The quest for homeostasis
Source: Plant Biotechnol J. 2023 Nov 30;22(2):427–44. doi: 10.1111/pbi.14196 (PMC10826984; doi:10.1111/pbi.14196)
Supplement: Supplementary file 12 — Figure S12 Transcriptional changes in the metabolic pathways of the ripe fruit of the β‐carotene line compared to the control. [file PBI-22-427-s006.pptx]

## Slide 1
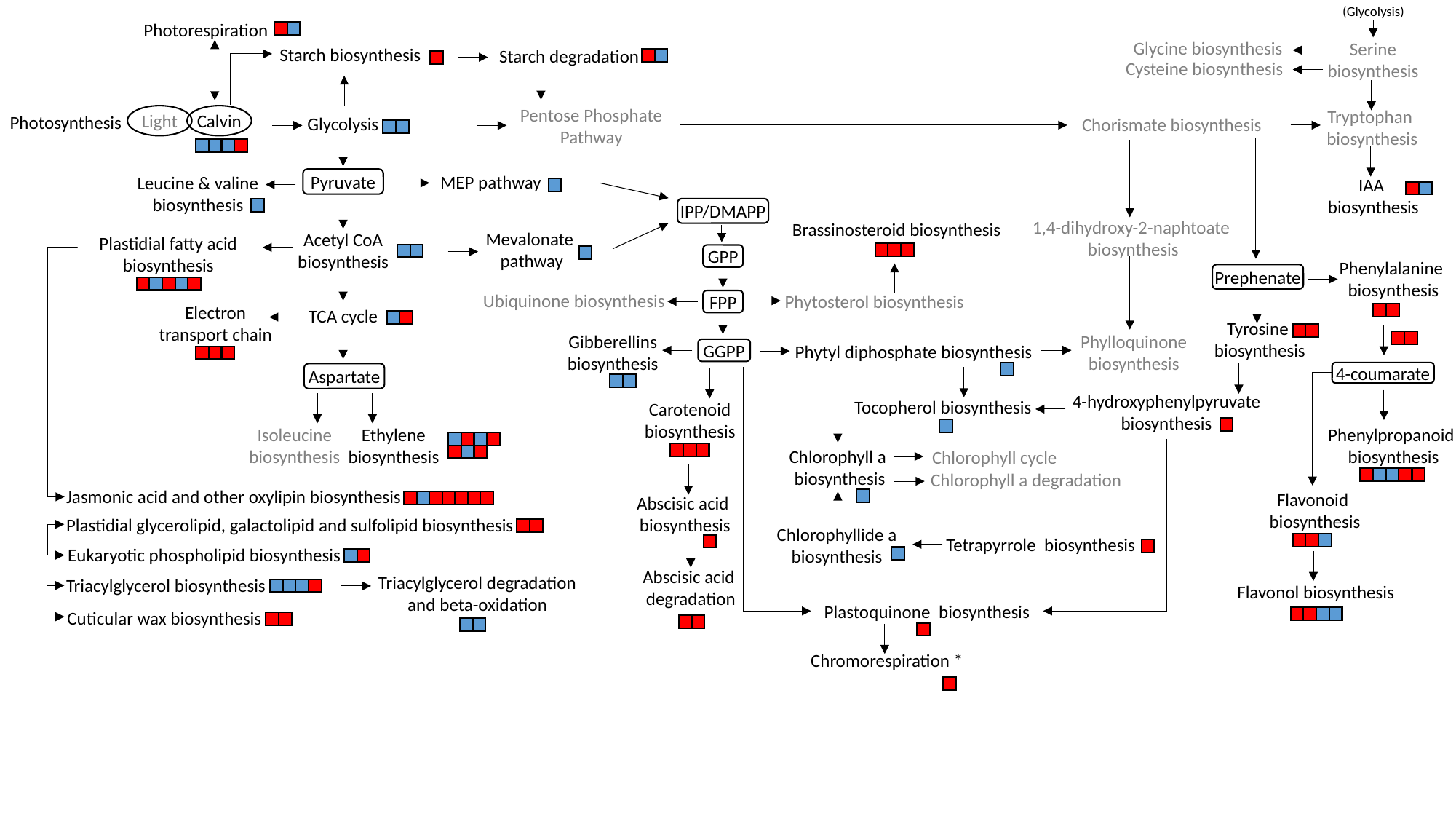

(Glycolysis)
Photorespiration
Glycine biosynthesis
Serine biosynthesis
Starch biosynthesis
Starch degradation
Cysteine biosynthesis
Pentose Phosphate Pathway
Tryptophan
 biosynthesis
Photosynthesis
Light
Calvin
Glycolysis
Chorismate biosynthesis
MEP pathway
Leucine & valine biosynthesis
IAA
 biosynthesis
Pyruvate
IPP/DMAPP
1,4-dihydroxy-2-naphtoate
 biosynthesis
Brassinosteroid biosynthesis
Mevalonate
 pathway
Acetyl CoA biosynthesis
Plastidial fatty acid biosynthesis
GPP
Phenylalanine
 biosynthesis
Prephenate
Ubiquinone biosynthesis
Phytosterol biosynthesis
FPP
Electron transport chain
TCA cycle
Tyrosine
 biosynthesis
Phylloquinone biosynthesis
Gibberellins
biosynthesis
Phytyl diphosphate biosynthesis
GGPP
4-coumarate
Aspartate
4-hydroxyphenylpyruvate biosynthesis
Tocopherol biosynthesis
Carotenoid biosynthesis
Phenylpropanoid
 biosynthesis
Isoleucine
biosynthesis
Ethylene
biosynthesis
Chlorophyll a
 biosynthesis
Chlorophyll cycle
Chlorophyll a degradation
Jasmonic acid and other oxylipin biosynthesis
Flavonoid
 biosynthesis
Abscisic acid
 biosynthesis
Plastidial glycerolipid, galactolipid and sulfolipid biosynthesis
Chlorophyllide a biosynthesis
Tetrapyrrole biosynthesis
Eukaryotic phospholipid biosynthesis
Abscisic acid
 degradation
Triacylglycerol degradation and beta-oxidation
Triacylglycerol biosynthesis
Flavonol biosynthesis
Plastoquinone biosynthesis
Cuticular wax biosynthesis
Chromorespiration *
