# Supplementary material for: Ketocarotenoid production in tomato triggers metabolic reprogramming and cellular adaptation: The quest for homeostasis
Source: Plant Biotechnol J. 2023 Nov 30;22(2):427–44. doi: 10.1111/pbi.14196 (PMC10826984; doi:10.1111/pbi.14196)
Supplement: Supplementary file 13 — Table S1 Carotenoid quantification in fruit over five ripening stages. [file PBI-22-427-s012.docx]

|  | **µg/g DW** | **25dpa** | | | **39dpa** | | | **43dpa** | | | **49dpa** | | | **66dpa** | | |
| --- | --- | --- | --- | --- | --- | --- | --- | --- | --- | --- | --- | --- | --- | --- | --- | --- |
| **CONTROL** | **Neo/viola** | 72.9 | ± | *10.1* | 63.7 | ± | *4.1* | 62.5 | ± | *5.8* | 0.0 | ± | *0.0* | 55.3 | ± | *1.1* |
|  | **Lutein** | 169.7 | ± | *15.2* | 172.2 | ± | *17.0* | 176.1 | ± | *2.4* | 161.8 | ± | *7.7* | 148.9 | ± | *1.0* |
|  | **cis-Lycopene** | 0.0 | ± | *0.0* | 0.0 | ± | *0.0* | 0.0 | ± | *0.0* | 76.5 | ± | *2.2* | 55.7 | ± | *2.5* |
|  | **Lycopene** | 0.0 | ± | *0.0* | 0.0 | ± | *0.0* | 35.1 | ± | *31.2* | 1903.3 | ± | *170.4* | 2644.0 | ± | *85.3* |
|  | **γ-Carotene** | 0.0 | ± | *0.0* | 0.0 | ± | *0.0* | 0.0 | ± | *0.0* | 71.0 | ± | *3.5* | 92.6 | ± | *2.7* |
|  | **β-Carotene** | 62.4 | ± | *1.4* | 64.1 | ± | *3.4* | 89.4 | ± | *12.9* | 164.3 | ± | *6.7* | 168.8 | ± | *8.2* |
|  | **Total carotenoids** | 305.0 | ± | 26.6 | 300.0 | ± | 22.3 | 363.2 | ± | 35.4 | 2376.9 | ± | 172.9 | 3165.3 | ± | 76.0 |
| **β-CAROTENE LINE** | **Neo/viola** | 84.5 | ± | *5.9* | 74.9 | ± | *3.8* | 65.8 | ± | *4.0* | 56.2 | ± | *3.2* | 59.0 | ± | *4.2* |
|  | **Lutein** | 180.0 | ± | *9.2* | 168.3 | ± | *10.9* | 154.2 | ± | *7.3* | 135.3 | ± | *8.6* | 131.0 | ± | *3.2* |
|  | **Lycopene** | 0.0 | ± | *0.0* | 0.0 | ± | *0.0* | 0.0 | ± | *0.0* | 59.6 | ± | *12.8* | 50.3 | ± | *3.8* |
|  | **α-carotene** | 0.0 | ± | *0.0* | 0.0 | ± | *0.0* | 0.0 | ± | *0.0* | 71.8 | ± | *6.3* | 105.8 | ± | *10.4* |
|  | **γ-Carotene** | 0.0 | ± | *0.0* | 0.0 | ± | *0.0* | 0.0 | ± | *0.0* | 94.4 | ± | *19.8* | 71.4 | ± | *11.8* |
|  | **β-Carotene** | 66.0 | ± | *1.2* | 62.8 | ± | *0.9* | 151.0 | ± | *73.7* | 954.8 | ± | *200.6* | 1014.9 | ± | *312.9* |
|  | **Total carotenoids** | 330.4 | ± | 16.2 | 306.1 | ± | 14.6 | 370.9 | ± | 78.5 | 1372.0 | ± | 215.9 | 1432.4 | ± | 329.0 |
| **KETOCAROTENOID LINE** | **Astaxanthin** | 53.1 | ± | *8.4* | 40.8 | ± | *9.6* | 25.5 | ± | *2.9* | 37.2 | ± | *6.5* | 76.3 | ± | *14.9* |
|  | **Phoenicoxanthin** | 26.1 | ± | *10.2* | 7.8 | ± | *11.5* | 49.1 | ± | *19.9* | 209.9 | ± | *16.2* | 518.9 | ± | *84.9* |
|  | **Canthaxanthin** | 22.0 | ± | *14.3* | 6.8 | ± | *9.3* | 132.9 | ± | *76.3* | 558.2 | ± | *111.5* | 801.6 | ± | *151.5* |
|  | **3'-OH-Echinenone** | 13.4 | ± | *1.0* | 12.4 | ± | *0.6* | 21.5 | ± | *6.6* | 78.5 | ± | *17.9* | 131.5 | ± | *33.9* |
|  | **Echinenone** | 14.1 | ± | *1.0* | 14.1 | ± | *0.4* | 19.8 | ± | *5.3* | 51.9 | ± | *5.9* | 92.5 | ± | *9.8* |
|  | **Phoenicoxanthin-C14:0** | 1.4 | ± | *0.0* | 1.5 | ± | *0.0* | 7.5 | ± | *6.0* | 128.2 | ± | *21.7* | 335.0 | ± | *187.6* |
|  | **Adonixanthin-C14:1** | 0.0 | ± | *0.0* | 0.0 | ± | *0.0* | 11.5 | ± | *4.5* | 44.8 | ± | *7.2* | 153.0 | ± | *63.9* |
|  | **Phoenicoxanthin-C16:0** | 2.2 | ± | *0.1* | 2.2 | ± | *0.2* | 21.2 | ± | *13.9* | 117.8 | ± | *22.5* | 203.3 | ± | *79.0* |
|  | **Adonixanthin-C16:1** | 7.6 | ± | *0.4* | 8.4 | ± | *0.2* | 15.6 | ± | *3.1* | 44.1 | ± | *7.3* | 86.3 | ± | *22.9* |
|  | **Adonixanthin epoxide** | 62.8 | ± | *4.7* | 63.6 | ± | *2.5* | 56.1 | ± | *3.0* | 0.0 | ± | *0.0* | 70.6 | ± | *0.8* |
|  | **Total ketocarotenoids** | 202.6 | ± | 21.6 | 157.5 | ± | 21.7 | 360.9 | ± | 134.6 | 1270.5 | ± | 197.0 | 2469.0 | ± | 572.0 |
|  | **Lutein** | 130.1 | ± | *1.7* | 126.6 | ± | *2.7* | 123.7 | ± | *2.0* | 0.0 | ± | *0.0* | 0.0 | ± | *0.0* |
|  | **β-Carotene** | 55.4 | ± | *1.0* | 62.5 | ± | *3.5* | 73.9 | ± | *12.1* | 169.3 | ± | *25.9* | 240.3 | ± | *75.5* |
|  | **Total carotenoids** | 185.4 | ± | 2.4 | 189.1 | ± | 5.3 | 197.6 | ± | 12.1 | 169.3 | ± | 25.9 | 240.3 | ± | 75.5 |
|  | **Total caro + keto** | 388.0 | ± | 23.4 | 346.7 | ± | 26.4 | 558.5 | ± | 145.4 | 1439.9 | ± | 173.2 | 2709.3 | ± | 540.6 |
|  | **% Phoenicoxanthin esterified/Total phoenico** | 12 | | | 32 | | | 37 | | | 54 | | | 51 | | |
|  |  |  |  |  |  |  |  |  |  |  |  |  |  |  |  |  |
