# Supplementary material for: Ketocarotenoid production in tomato triggers metabolic reprogramming and cellular adaptation: The quest for homeostasis
Source: Plant Biotechnol J. 2023 Nov 30;22(2):427–44. doi: 10.1111/pbi.14196 (PMC10826984; doi:10.1111/pbi.14196)
Supplement: Supplementary file 14 — Table S2 Electron microscopy related measurements of mature green and ripe tomato fruit electron micrographs. [file PBI-22-427-s022.docx]

A

| **MG** | | **% of plastids containing X membranous sacs** | | | | | | | | | |  | | |  |  |  |  |  |  |  |  |  |  |  |
| --- | --- | --- | --- | --- | --- | --- | --- | --- | --- | --- | --- | --- | --- | --- | --- | --- | --- | --- | --- | --- | --- | --- | --- | --- | --- |
| **X=** | | **≤10** | | | | | **>10** | | | | | **total %** | | |  |  |  |  |  |  |  |  |  |  |  |
| **Control** | | 32 | | | | | 6 | | | | | 37 | | |  |  |  |  |  |  |  |  |  |  |  |
| **β-caro line** | | 22 | | | | | 6 | | | | | 28 | | |  |  |  |  |  |  |  |  |  |  |  |
| **Keto line** | | 19 | | | | | **47**** | | | | | **66*** | | |  |  |  |  |  |  |  |  |  |  |  |
|  | |  | | | | |  | | | | |  | | |  |  |  |  |  |  |  |  |  |  |  |
| **RIPE** | | **% of plastids with containing X membranous sacs** | | | | | | | | | |  | | |  |  |  |  |  |  |  |  |  |  |  |
| **X=** | | **≤10** | | | | | **>10** | | | | | **total %** | | |  |  |  |  |  |  |  |  |  |  |  |
| **Control** | | 0 | | | | | 0 | | | | | 0 | | |  |  |  |  |  |  |  |  |  |  |  |
| **β-caro line** | | 0 | | | | | 0 | | | | | 0 | | |  |  |  |  |  |  |  |  |  |  |  |
| **Keto line** | | **57***** | | | | | 0 | | | | | **57***** | | |  |  |  |  |  |  |  |  |  |  |  |
| B |  | |  |  |  |  | |  |  |  |  | |  |  | |  | **Grey value (colour intensity)** | | | | | | | | |
| **MG** | **Plastid area (µm^2^)** | | | | **Number of PG** | | | | **total PG area** | | | | **Plastid/PG area** | | | | **Average PG** | | | **Background** | | | **Ratio** | | |
| **Control** | 5.5 | | ± | 1.9 | 16 | ± | | 7 | 0.2 | ± | 0.2 | | 49.4 | ± | | 39.3 | 78.1 | ± | 19.6 | 189.9 | ± | 15.2 | 2.5 | ± | 0.5 |
| **β-caro line** | **4.1** | | **±** | **1.5*** | 19 | ± | | 12 | 0.1 | ± | 0.1 | | 42.8 | ± | | 19.8 | **111.1** | **±** | **27.5**** | 189.8 | ± | 23.7 | **1.8** | **±** | **0.3***** |
| **Keto line** | 4.9 | | ± | 2.2 | 17 | ± | | 10 | 0.2 | ± | 0.2 | | 30.9 | ± | | 14.9 | **61.7** | **±** | **13.9*** | 175.8 | ± | 18.2 | 3.0 | ± | 0.7 |
|  |  | |  |  |  |  | |  |  |  |  | |  |  | |  |  |  |  |  |  |  |  |  |  |
|  |  | |  |  |  |  | |  |  |  |  | |  |  | |  | **Grey value (colour intensity)** | | | | | | | | |
| **RIPE** | **Plastid area (µm^2^)** | | | | **Number of PG** | | | | **total PG area** | | | | **Plastid/PG area** | | | | **Average PG** | | | **Background** | | | **Ratio** | | |
| **Control** | 2.0 | | ± | 0.8 | 8 | ± | | 4 | 0.3 | ± | 0.1 | | 8.4 | ± | | 4.9 | 54.3 | ± | 11.4 | 114.0 | ± | 6.1 | 2.2 | ± | 0.4 |
| **β-caro line** | **6.7** | | **±** | **2.3*** | 14 | ± | | 8 | 1.2 | ± | 0.6 | | 6.1 | ± | | 1.1 | **108.5** | **±** | **12.4***** | **144.3** | **±** | **5.6*** | 1.3 | ± | 0.2 |
| **Keto line** | **6.3** | | **±** | **2.6**** | 14 | ± | | 6 | **1.6** | **±** | **0.6**** | | 4.1 | ± | | 0.8 | **43.9** | **±** | **6.6*** | **185.2** | **±** | **17.0***** | **4.4** | **±** | **0.6***** |
|  |  | |  |  |  |  | |  |  |  |  | |  |  | |  | *Grey value range: black = 0, white = 255* | | | | | | |  |  |
