# Supplementary material for: Ketocarotenoid production in tomato triggers metabolic reprogramming and cellular adaptation: The quest for homeostasis
Source: Plant Biotechnol J. 2023 Nov 30;22(2):427–44. doi: 10.1111/pbi.14196 (PMC10826984; doi:10.1111/pbi.14196)
Supplement: Supplementary file 15 — Table S3 Carotenoid quantification of the fruit chromoplast fractions. [file PBI-22-427-s003.docx]

| **% of µg/fraction** |  |  |  |  |  |  |  |  |  |  |  |  |  |  |  |  |  |  |  |  |  |  |  |  |  |  |  |  |
| --- | --- | --- | --- | --- | --- | --- | --- | --- | --- | --- | --- | --- | --- | --- | --- | --- | --- | --- | --- | --- | --- | --- | --- | --- | --- | --- | --- | --- |
|  |  |  |  |  |  |  |  |  |  |  |  |  |  |  |  |  |  |  |  |  |  |  |  |  |  |  |  |  |
| **Control** | **F1** | **F2** | **F3** | **F4** | **F5** | **F6** | **F7** | **F8** | **F9** | **F10** | **F11** | **F12** | **F13** | **F14** | **F15** | **F16** | **F17** | **F18** | **F19** | **F20** | **F21** | **F22** | **F23** | **F24** | **F25** | **F26** | **F27** | **F28** |
| **Lycopene** | 2.6 | 2.6 | 2.4 | 4.0 | 3.4 | 4.4 | 3.5 | 4.0 | 2.7 | 1.7 | 2.4 | 1.6 | 2.3 | 3.0 | 5.0 | 5.9 | 10.7 | 16.2 | 6.1 | 4.5 | 3.7 | 1.7 | 1.6 | 0.9 | 0.9 | 0.9 | 0.6 | 0.8 |
| **Lutein** | 3.5 | 3.5 | 3.5 | 3.5 | 3.5 | 3.5 | 3.5 | 3.5 | 3.5 | 3.5 | 3.5 | 3.5 | 3.5 | 3.5 | 3.6 | 3.8 | 4.1 | 4.1 | 3.6 | 3.5 | 3.6 | 3.5 | 3.5 | 3.5 | 3.5 | 3.5 | 3.5 | 3.5 |
| **β-Carotene** | 3.4 | 3.2 | 3.1 | 3.2 | 3.1 | 3.2 | 3.1 | 3.2 | 3.0 | 2.9 | 3.0 | 2.9 | 3.0 | 3.2 | 3.5 | 3.7 | 5.2 | 8.9 | 4.7 | 4.4 | 3.9 | 3.5 | 3.4 | 3.2 | 3.2 | 3.1 | 3.0 | 3.0 |
| **Phytofluene** | 4.9 | 3.7 | 3.0 | 3.1 | 2.7 | 2.8 | 2.7 | 2.9 | 2.7 | 2.5 | 2.8 | 2.7 | 3.0 | 3.9 | 4.9 | 5.1 | 6.5 | 7.5 | 4.9 | 4.4 | 3.9 | 3.5 | 3.0 | 2.7 | 2.6 | 2.5 | 2.3 | 2.4 |
| **Carotenoids** | 3.1 | 2.9 | 2.7 | 3.8 | 3.3 | 4.0 | 3.4 | 3.8 | 2.8 | 2.1 | 2.6 | 2.1 | 2.6 | 3.2 | 4.7 | 5.3 | 8.9 | 13.0 | 5.5 | 4.4 | 3.7 | 2.3 | 2.2 | 1.6 | 1.6 | 1.6 | 1.4 | 1.5 |
| **Cyclic carotenoids** | 3.5 | 3.4 | 3.4 | 3.4 | 3.4 | 3.4 | 3.4 | 3.4 | 3.3 | 3.3 | 3.3 | 3.3 | 3.3 | 3.4 | 3.5 | 3.8 | 4.5 | 5.8 | 4.0 | 3.8 | 3.7 | 3.5 | 3.5 | 3.4 | 3.4 | 3.4 | 3.3 | 3.3 |
|  |  |  |  |  |  |  |  |  |  |  |  |  |  |  |  |  |  |  |  |  |  |  |  |  |  |  |  |  |
|  |  |  |  |  |  |  |  |  |  |  |  |  |  |  |  |  |  |  |  |  |  |  |  |  |  |  |  |  |
| **Keto line** | **F1** | **F2** | **F3** | **F4** | **F5** | **F6** | **F7** | **F8** | **F9** | **F10** | **F11** | **F12** | **F13** | **F14** | **F15** | **F16** | **F17** | **F18** | **F19** | **F20** | **F21** | **F22** | **F23** | **F24** | **F25** | **F26** | **F27** | **F28** |
| **Astaxanthin** | 1.8 | 0.6 | 1.0 | 1.2 | 1.0 | 1.0 | 0.8 | 0.8 | 1.1 | 0.8 | 2.0 | 2.5 | 3.3 | 5.1 | 7.8 | 7.5 | 17.7 | 34.5 | 5.1 | 2.3 | 1.3 | 0.4 | 0.1 | 0.0 | 0.0 | 0.0 | 0.0 | 0.1 |
| **Phoenicoxanthin** | 4.3 | 1.9 | 2.0 | 1.9 | 1.5 | 1.2 | 1.2 | 1.1 | 1.6 | 0.8 | 1.5 | 1.8 | 2.4 | 3.8 | 5.7 | 6.1 | 16.9 | 34.6 | 5.0 | 2.3 | 1.3 | 0.4 | 0.2 | 0.0 | 0.0 | 0.0 | 0.0 | 0.2 |
| **Canthaxanthin** | 5.1 | 2.7 | 2.7 | 2.5 | 2.2 | 1.6 | 1.7 | 1.6 | 2.1 | 1.2 | 1.6 | 1.8 | 2.3 | 3.4 | 5.0 | 5.5 | 14.8 | 27.7 | 5.2 | 2.9 | 1.9 | 1.1 | 0.8 | 0.5 | 0.5 | 0.4 | 0.5 | 0.9 |
| **3ˈOH-Echinenone** | 5.3 | 3.0 | 3.2 | 3.2 | 2.6 | 2.1 | 2.4 | 2.2 | 2.3 | 1.9 | 2.1 | 2.1 | 2.6 | 3.6 | 5.1 | 4.7 | 11.9 | 23.9 | 4.0 | 2.4 | 1.9 | 1.3 | 1.2 | 1.0 | 1.0 | 0.9 | 0.9 | 1.2 |
| **3OH-Echinenone** | 2.7 | 2.6 | 3.9 | 3.7 | 3.4 | 2.9 | 2.9 | 2.7 | 3.4 | 2.8 | 2.5 | 2.8 | 3.1 | 3.6 | 4.1 | 4.3 | 8.5 | 15.4 | 4.8 | 3.7 | 2.3 | 2.3 | 2.1 | 2.0 | 2.0 | 2.0 | 1.9 | 2.0 |
| **Echinenone** | 3.6 | 2.6 | 2.6 | 2.7 | 2.7 | 2.1 | 2.3 | 2.2 | 2.6 | 1.9 | 2.3 | 2.5 | 2.8 | 3.4 | 4.5 | 4.3 | 12.5 | 24.4 | 4.4 | 2.6 | 1.9 | 1.5 | 1.4 | 1.2 | 1.2 | 1.2 | 1.2 | 1.4 |
| **Phoenicoxanthin-C14:0** | 5.1 | 2.2 | 2.1 | 1.9 | 1.4 | 0.7 | 0.8 | 0.8 | 1.3 | 0.3 | 0.7 | 1.0 | 1.8 | 3.1 | 5.3 | 5.5 | 17.0 | 39.5 | 5.4 | 2.4 | 1.2 | 0.2 | 0.1 | 0.0 | 0.0 | 0.0 | 0.0 | 0.0 |
| **Adonixanthin-C14:1** | 5.0 | 2.7 | 2.9 | 2.6 | 2.3 | 1.6 | 1.9 | 1.8 | 2.2 | 1.6 | 1.7 | 1.9 | 2.5 | 3.5 | 4.9 | 4.7 | 13.0 | 28.6 | 4.7 | 2.3 | 1.8 | 1.0 | 0.9 | 0.7 | 0.7 | 0.6 | 1.3 | 0.9 |
| **Phoenicoxanthin-C16:0** | 5.5 | 2.5 | 2.4 | 2.1 | 1.7 | 0.8 | 0.9 | 0.9 | 1.5 | 0.1 | 0.6 | 1.0 | 1.6 | 2.9 | 5.0 | 5.3 | 17.7 | 38.4 | 5.8 | 2.3 | 1.0 | 0.1 | 0.0 | 0.0 | 0.0 | 0.0 | 0.0 | 0.0 |
| **Adonixanthin-C16:1** | 5.0 | 2.6 | 2.8 | 2.8 | 2.4 | 1.5 | 1.8 | 1.9 | 2.1 | 1.4 | 1.8 | 1.8 | 2.3 | 3.1 | 4.5 | 4.1 | 10.9 | 24.5 | 9.7 | 6.5 | 1.6 | 1.1 | 0.8 | 0.5 | 0.6 | 0.5 | 0.5 | 0.8 |
| **Adonixanthin epoxide** | 3.5 | 3.4 | 3.4 | 3.4 | 3.4 | 3.4 | 3.4 | 3.4 | 3.4 | 3.4 | 3.5 | 3.5 | 3.5 | 3.7 | 3.9 | 3.9 | 4.6 | 5.5 | 3.7 | 3.5 | 3.4 | 3.4 | 3.3 | 3.3 | 3.3 | 3.3 | 3.3 | 3.3 |
| **Free ketocarotenoids** | 4.7 | 2.5 | 2.6 | 2.5 | 2.1 | 1.6 | 1.7 | 1.6 | 2.0 | 1.3 | 1.7 | 1.9 | 2.4 | 3.5 | 5.2 | 5.5 | 14.8 | 28.4 | 5.0 | 2.7 | 1.8 | 1.0 | 0.8 | 0.5 | 0.5 | 0.4 | 0.5 | 0.8 |
| **Ketocartenoid esters** | 5.2 | 2.4 | 2.4 | 2.2 | 1.8 | 0.9 | 1.1 | 1.1 | 1.6 | 0.5 | 0.9 | 1.2 | 1.9 | 3.1 | 5.0 | 5.2 | 15.9 | 35.7 | 6.0 | 2.9 | 1.3 | 0.4 | 0.3 | 0.2 | 0.2 | 0.2 | 0.2 | 0.2 |
| **β-Carotene** | 2.2 | 2.4 | 2.5 | 2.7 | 2.8 | 2.2 | 2.3 | 2.2 | 2.4 | 2.0 | 2.3 | 2.5 | 2.9 | 3.5 | 4.5 | 4.0 | 12.4 | 24.0 | 4.4 | 3.1 | 1.9 | 1.8 | 1.6 | 1.5 | 1.5 | 1.4 | 1.7 | 1.5 |
|  |  |  |  |  |  |  |  |  |  |  |  |  |  |  |  |  |  |  |  |  |  |  |  |  |  |  |  |  |
|  |  |  |  |  |  |  |  |  |  |  |  |  |  |  |  |  |  |  |  |  |  |  |  |  |  |  |  |  |
| **β-caro line** | **F1** | **F2** | **F3** | **F4** | **F5** | **F6** | **F7** | **F8** | **F9** | **F10** | **F11** | **F12** | **F13** | **F14** | **F15** | **F16** | **F17** | **F18** | **F19** | **F20** | **F21** | **F22** | **F23** | **F24** | **F25** | **F26** | **F27** | **F28** |
| **Neo/Violaxanthin** | 0.0 | 0.0 | 0.0 | 0.0 | 3.8 | 3.8 | 3.8 | 3.9 | 3.9 | 3.9 | 3.9 | 4.0 | 4.2 | 4.3 | 4.9 | 5.0 | 4.9 | 5.6 | 4.5 | 4.2 | 3.9 | 4.0 | 3.9 | 3.9 | 3.9 | 3.9 | 3.9 | 4.0 |
| **Lutein** | 3.5 | 3.5 | 3.5 | 3.5 | 3.5 | 3.5 | 3.5 | 3.5 | 3.5 | 3.5 | 3.5 | 3.5 | 3.6 | 3.6 | 3.8 | 3.8 | 3.9 | 4.2 | 3.8 | 3.6 | 3.5 | 3.5 | 3.5 | 3.5 | 3.5 | 3.5 | 3.5 | 3.5 |
| **Lycopene** | 0.0 | 0.0 | 0.0 | 3.4 | 3.5 | 3.8 | 3.9 | 3.8 | 3.7 | 3.6 | 3.7 | 3.7 | 3.7 | 3.7 | 4.0 | 4.1 | 4.8 | 10.6 | 5.4 | 3.7 | 3.5 | 3.4 | 3.4 | 3.3 | 3.3 | 3.3 | 3.3 | 3.3 |
| **α-Carotene** | 3.4 | 3.4 | 3.4 | 3.5 | 3.5 | 3.6 | 3.6 | 3.7 | 3.6 | 3.6 | 3.6 | 3.6 | 3.6 | 3.6 | 3.6 | 3.6 | 3.8 | 5.0 | 3.9 | 3.5 | 3.4 | 3.4 | 3.4 | 3.4 | 3.3 | 3.3 | 3.3 | 3.4 |
| **γ-Carotene** | 0.0 | 3.3 | 3.6 | 3.6 | 3.5 | 3.5 | 3.4 | 3.3 | 3.3 | 3.3 | 3.4 | 3.2 | 3.2 | 3.3 | 3.4 | 3.8 | 4.4 | 10.0 | 5.1 | 3.6 | 3.4 | 3.3 | 3.2 | 3.2 | 3.2 | 3.2 | 3.2 | 3.2 |
| **β-Carotene** | 1.9 | 2.4 | 4.1 | 4.3 | 3.6 | 3.5 | 3.4 | 3.1 | 2.5 | 1.9 | 2.3 | 2.3 | 2.3 | 2.2 | 3.5 | 3.7 | 5.8 | 24.3 | 8.6 | 2.9 | 2.0 | 1.8 | 1.3 | 1.3 | 1.2 | 1.1 | 1.2 | 1.6 |
| **Carotenoids** | 1.9 | 2.4 | 3.2 | 3.6 | 3.5 | 3.6 | 3.5 | 3.4 | 3.1 | 2.8 | 3.0 | 3.0 | 3.0 | 3.0 | 3.7 | 3.9 | 4.9 | 14.7 | 6.3 | 3.3 | 2.8 | 2.7 | 2.5 | 2.4 | 2.4 | 2.4 | 2.4 | 2.6 |
| **Cyclic carotenoids** | 1.9 | 2.5 | 3.5 | 3.6 | 3.6 | 3.5 | 3.4 | 3.3 | 2.9 | 2.6 | 2.9 | 2.9 | 2.9 | 2.9 | 3.7 | 3.9 | 5.1 | 16.2 | 6.7 | 3.3 | 2.7 | 2.6 | 2.3 | 2.3 | 2.2 | 2.2 | 2.2 | 2.5 |
