# Supplementary material for: Ketocarotenoid production in tomato triggers metabolic reprogramming and cellular adaptation: The quest for homeostasis
Source: Plant Biotechnol J. 2023 Nov 30;22(2):427–44. doi: 10.1111/pbi.14196 (PMC10826984; doi:10.1111/pbi.14196)
Supplement: Supplementary file 20 — Table S8 Expression data of plastidial redox regulatory genes. [file PBI-22-427-s007.docx]

| **Plastidial redox regulation genes** | | **Fold change** | | |
| --- | --- | --- | --- | --- |
|  |  | **Keto/Control** | **Keto/β-caro** | **β-caro/Control** |
| *Solyc04g057980* | NAD(P)H-quinone oxidoreductase subunit M, chloroplastic | 19.7 | 11.2 | NS |
| *Solyc05g026550* | NAD(P)H-quinone oxidoreductase subunit L, chloroplastic | 4.9 | 4.9 | NS |
| *Solyc09g083150* | NAD(P)H-quinone oxidoreductase subunit N, chloroplastic | 2.3 | 1.8 | NS |
| *Solyc01g096240* | NADH-plastoquinone oxidoreductase subunit 5 (chloroplast) | 1.6 | 2.3 | NS |
| *Solyc08g081690* | NADPH oxidase | 2.6 | NS | NS |
| *Solyc09g091070* | Malate dehydrogenase, chloroplastic | 1.6 | 1.8 | NS |
| *Solyc03g115005* | PsbQ-like protein 3, chloroplastic | 14.9 | NS | NS |
| *Solyc12g005630* | Cytochrome b6-f complex iron-sulfur subunit, chloroplastic | 2.1 | 1.7 | NS |
| *Solyc08g080050* | PGR5-like protein 1A, chloroplastic | 1.7 | NS | NS |
| *Solyc12g009400* | Pyruvate dehydrogenase E1 component subunit alpha-3, chloroplastic | 1.7 | 2.3 | NS |
| *Solyc09g083190* | Photosynthetic NDH subunit of subcomplex B 5, chloroplastic | 7.5 | 8.9 | NS |
| *Solyc05g007780* | Photosynthetic NDH subcomplex L 2 | 3.5 | 4.4 | NS |
| *Solyc02g079750* | Probable NAD(P)H dehydrogenase (quinone) FQR1-like 2 | 1.6 | NS | NS |
| *Solyc03g034140* | Probable NAD(P)H dehydrogenase (quinone) FQR1-like 3 | 0.3 | NS | 0.4 |
| *Solyc10g082030* | 2-Cys peroxiredoxin 1 | 1.6 | NS | NS |
| *Solyc02g083810* | Ferredoxin--NADP reductase, leaf-type isozyme, chloroplastic | 2.3 | 2.0 | NS |
| *Solyc09g007190* | Thioredoxin-like protein AAED1, chloroplastic isoform X1 | 2 | 2.8 | NS |
| *Solyc03g115870* | Thioredoxin-like 1-2, chloroplastic | 0.5 | NS | 0.4 |
| *Solyc04g078910* | Thioredoxin-like 4, chloroplastic | 2.8 | NS | NS |
