# Supplementary material for: Ketocarotenoid production in tomato triggers metabolic reprogramming and cellular adaptation: The quest for homeostasis
Source: Plant Biotechnol J. 2023 Nov 30;22(2):427–44. doi: 10.1111/pbi.14196 (PMC10826984; doi:10.1111/pbi.14196)
Supplement: Supplementary file 26 — Table S14 Chromatographic details of compounds analysed by UPLC. [file PBI-22-427-s015.docx]

|  | **UPLC method** | **Spectrum** | **RT (min)** |
| --- | --- | --- | --- |
| **Neoxanthin** | Nogueira et al 2017 | 414.1, 438.3, 467.4 | 2.3 |
| **Violaxanthin** | Nogueira et al 2017 | 417.7, 442.0, 471.1 | 2.5 |
| **Adonixanthin epoxide** | Nogueira et al 2017 | 454.1 473.5 | 2.9 |
| **Astaxanthin** | Nogueira et al 2017 | 479.6 | 3.2 |
| **Adonixanthin** | Nogueira et al 2017 | 466.2 | 3.9 |
| **Zeaxanthin** | Nogueira et al 2017 | -, 454.1, 480.8 | 4.2 |
| **Lutein** | Nogueira et al 2017 | -, 448.0, 476.0 | 4.3 |
| **Phoenicoxanthin** | Nogueira et al 2017 | 479.6 | 4.6 |
| **Canthaxanthin** | Nogueira et al 2017 | 477.2 | 5.9 |
| **3'-OH-Echinenone** | Nogueira et al 2017 | 465 | 6.2 |
| **Cis-lycopene** | Nogueira et al 2017 | 294.6, - , 472.3, 502.7 | 6.9 |
| **Chlorophyll b** | Nogueira et al 2017 | 456.5 | 6.9 |
| **Echinenone** | Nogueira et al 2017 | 460.2 | 7.0 |
| **α-Tocopherol** | Nogueira et al 2017 | 293.4 | 7.1 |
| **Chlorophyll a** | Nogueira et al 2017 | 429.8 | 7.2 |
| **Lycopene** | Nogueira et al 2017 | -, 472.3, 502.7 | 7.3 |
| **Phoenicoxanthin-C14:0** | Nogueira et al 2017 | 476 | 7.4 |
| **Adonixanthin-C14:1** | Nogueira et al 2017 | 466.2 | 7.5 |
| **γ-carotene** | Nogueira et al 2017 | -, 462.6, 493.0 | 7.8 |
| **Phoenicoxanthin-C16:0** | Nogueira et al 2017 | 476 | 7.8 |
| **Adonixanthin-C16:1** | Nogueira et al 2017 | 466.2 | 7.9 |
| **β-Carotene** | Nogueira et al 2017 | -, 454.1, 479.6 | 8.1 |
| **Plastoquinone** | Nogueira et al 2013 | 254.3, - | 7.3 |
| **Putative astaxanthin di-esters 1** | this paper | 473.5 | 9 |
| **Putative astaxanthin di-esters 2** | this paper | 476 | 9.8 |
| **Putative astaxanthin di-esters 3** | this paper | 478.4 | 10.8 |

**Table S14: Chromatographic details of compounds analysed by UPLC**
